# Supplementary material for: Multilayer MEG source modelling enables depth-resolved laminar inference in humans
Source: Nat Commun. 2026 Sep 30;17:10128. doi: 10.1038/s41467-026-77782-9 (PMC13627721; doi:10.1038/s41467-026-77782-9)
Supplement: Supplementary file 2 [file 41467_2026_77782_MOESM2_ESM.pdf]

nature portfolio

Corresponding author(s): James J Bonaiuto

Last updated by author(s): Jul 30, 2026

# Reporting Summary

Nature Portfolio wishes to improve the reproducibility of the work that we publish. This form provides structure for consistency and transparency in reporting. For further information on Nature Portfolio policies, see our Editorial Policies and the Editorial Policy Checklist.

## Statistics

For all statistical analyses, confirm that the following items are present in the figure legend, table legend, main text, or Methods section.

| n/a | Confirmed | |
|---|---|---|
| ☐ | ☒ | The exact sample size (*n*) for each experimental group/condition, given as a discrete number and unit of measurement |
| ☐ | ☒ | A statement on whether measurements were taken from distinct samples or whether the same sample was measured repeatedly |
| ☐ | ☒ | The statistical test(s) used AND whether they are one- or two-sided<br>*Only common tests should be described solely by name; describe more complex techniques in the Methods section.* |
| ☐ | ☒ | A description of all covariates tested |
| ☐ | ☒ | A description of any assumptions or corrections, such as tests of normality and adjustment for multiple comparisons |
| ☐ | ☒ | A full description of the statistical parameters including central tendency (e.g. means) or other basic estimates (e.g. regression coefficient) AND variation (e.g. standard deviation) or associated estimates of uncertainty (e.g. confidence intervals) |
| ☐ | ☒ | For null hypothesis testing, the test statistic (e.g. *F*, *t*, *r*) with confidence intervals, effect sizes, degrees of freedom and *P* value noted<br>*Give P values as exact values whenever suitable.* |
| ☐ | ☒ | For Bayesian analysis, information on the choice of priors and Markov chain Monte Carlo settings |
| ☐ | ☒ | For hierarchical and complex designs, identification of the appropriate level for tests and full reporting of outcomes |
| ☐ | ☒ | Estimates of effect sizes (e.g. Cohen's *d*, Pearson's *r*), indicating how they were calculated |

*Our web collection on statistics for biologists contains articles on many of the points above.*

## Software and code

Policy information about availability of computer code

| | |
|---|---|
| Data collection | MEG and MRI data were collected as part of previously published studies using standard acquisition software provided by the respective scanner manufacturers. Experimental stimulus presentation and behavioral response collection were performed using task-specific software available at: https://github.com/jbonaiuto/cued_action_selection, https://github.com/danclab/auditory_laminar, and https://gitlab.com/TommyClausner/meglyon-experiment-runner. |
| Data analysis | Data analysis was performed using a combination of custom and open-source software. Structural MRI data were processed using FreeSurfer v6.0 for cortical surface reconstruction. MEG source reconstruction and Bayesian model comparison were performed using laMEG v0.0.7 (10.5281/zenodo.21638150) including a customized version of SPM12, the Variational Bayesian Analysis (VBA) toolbox (v5899497), and OpenMEEG v2.1.0. Custom analysis code used in this study is publicly available at: https://github.com/danclab/multilaminar_sim/, https://github.com/danclab/laminar_motor_erf/, https://github.com/danclab/auditory_laminar, and https://github.com/cophyteam/laminar_visual_erf. |

For manuscripts utilizing custom algorithms or software that are central to the research but not yet described in published literature, software must be made available to editors and reviewers. We strongly encourage code deposition in a community repository (e.g. GitHub). See the Nature Portfolio guidelines for submitting code & software for further information.

# Data

Policy information about availability of data

All manuscripts must include a data availability statement. This statement should provide the following information, where applicable:

- Accession codes, unique identifiers, or web links for publicly available datasets
- A description of any restrictions on data availability
- For clinical datasets or third party data, please ensure that the statement adheres to our policy

The preprocessed MEG data, structural MRI scans, cortical surface reconstructions, and fiducial locations underlying the empirical analyses have been deposited in the Zenodo repository (https://doi.org/10.5281/zenodo.20721185). Raw MEG and MRI data are not publicly available because they contain identifiable human participant data and are subject to institutional ethical and data protection requirements. Access to the raw data may be granted to qualified researchers for research purposes, subject to approval by the relevant ethics committee and completion of an appropriate data-sharing agreement. Requests should be directed to the corresponding author, who will respond within a reasonable timeframe. Access will be provided for the duration of the approved research project. Source data are provided with this paper.

# Research involving human participants, their data, or biological material

Policy information about studies with human participants or human data. See also policy information about sex, gender (identity/presentation), and sexual orientation and race, ethnicity and racism.

| | |
|---|---|
| Reporting on sex and gender | Participant sex was recorded for all datasets based on participant self-report. The study was not designed or powered to investigate sex-related differences in laminar source reconstruction, which was the primary methodological objective. Accordingly, no formal analyses stratified by sex were performed. Information on gender identity was not collected. Total participants: 41 (23 female, 18 male). |
| Reporting on race, ethnicity, or other socially relevant groupings | No race, ethnicity, or other socially relevant grouping variables were collected or used in this study. Consequently, participants were not classified into any such categories, and no analyses based on race, ethnicity, or other socially relevant groupings were performed. Because these variables were neither collected nor included in the study design, no controls for potential confounding effects of race, ethnicity, or other socially relevant groupings were applied. |
| Population characteristics | Participants were healthy adult volunteers recruited as part of three independent MEG studies. The study included 41 participants in total (18 male, 23 female). Dataset-specific mean ages were 25.7 ± 3.9 years (visual dataset), 28.5 ± 8.5 years (visually cued button-press dataset), and 32.4 ± 5.9 years (self-paced button-press dataset). Participants were free of neurological and psychiatric disorders according to the inclusion criteria of the original studies. Participants in the visual dataset were right-handed with normal or corrected-to-normal vision. No genotypic information was collected, no treatment groups were assigned, and no clinical diagnoses were investigated. |
| Recruitment | Participants were recruited from the local university community, primarily through advertisements, flyers, and word of mouth. Participation was voluntary. As with many cognitive neuroscience studies based on volunteer recruitment, the sample may be subject to self-selection bias, including overrepresentation of individuals who are willing and able to participate in research studies and who are affiliated with or live near a university environment. Because the primary aim of this study was methodological evaluation of MEG-based laminar inference rather than estimation of population-level behavioral or clinical effects, such biases are unlikely to substantially affect the principal conclusions. Participants in the visually evoked response dataset received €40 for participation. Participants in the visually cued button-press dataset received £60–200, depending on the number of MRI and MEG sessions completed. Participants in the self-paced button-press dataset did not receive financial compensation. |
| Ethics oversight | The visually evoked response dataset was approved by the Comité de Protection des Personnes Est IV (CPP Est IV; protocol 2019-A01604-53). The visually cued button-press dataset was approved by the University College London Research Ethics Committee (reference 5833/001). The self-paced button-press dataset was approved by the Comité de Protection des Personnes Sud-Méditerranée V (CPP Sud-Méditerranée V; protocol 2021-A00042-39). All participants provided written informed consent, and all procedures were conducted in accordance with the Declaration of Helsinki. |

Note that full information on the approval of the study protocol must also be provided in the manuscript.

# Field-specific reporting

Please select the one below that is the best fit for your research. If you are not sure, read the appropriate sections before making your selection.

☒ Life sciences ☐ Behavioural & social sciences ☐ Ecological, evolutionary & environmental sciences

For a reference copy of the document with all sections, see nature.com/documents/nr-reporting-summary-flat.pdf

# Life sciences study design

All studies must disclose on these points even when the disclosure is negative.

| | |
|---|---|
| Sample size | No formal statistical power calculation was performed. The empirical analyses were conducted using three previously collected MEG datasets, and sample sizes were determined by the number of participants available in those datasets after application of predefined inclusion criteria. |

These sample sizes are comparable to those used in previous high-precision MEG and laminar MEG studies. Because the primary aim of the study was methodological development and validation of a multilayer source reconstruction framework, the robustness of the findings was assessed through extensive simulations, replication across independent visual and sensorimotor datasets, and group-level Bayesian model comparison. The consistency of the observed laminar patterns across participants and datasets indicates that the available sample sizes were sufficient for the objectives of the study.

| | |
|---|---|
| Data exclusions | Data exclusions were based on a predefined sensor-level signal-to-noise ratio (SNR) criterion. Because laminar inference was performed on trial-averaged event-related fields (ERFs), sensor-level SNR was used as the primary measure of data quality. Participants whose SNR within the component-specific analysis window was below -15 dB were excluded from further analysis. This conservative threshold was established a priori based on simulation results demonstrating that laminar inference becomes unreliable below this SNR level. Applying this criterion resulted in the exclusion of 6 participants from the visually evoked response dataset and no participants from either the visually cued button-press or self-paced button-press datasets. No additional exclusions were performed. |
| Replication | Simulation experiments were repeated across large numbers of cortical locations under multiple independent conditions, including varying signal-to-noise ratio, co-registration error, noise structure, forward-model mismatch, source-space mismatch, and concurrent source activity. Empirical findings were evaluated independently in three separate human MEG datasets (28, 8, and 5 participants, respectively), with the two motor datasets providing independent replication of sensorimotor laminar activation patterns. All planned replication and robustness analyses were successful and supported the principal conclusions of the study; no planned analyses failed to replicate. |
| Randomization | Randomization was not relevant to this study. Participants were not allocated to experimental groups for the purposes of the present analyses; rather, previously collected MEG datasets were reanalyzed using the proposed multilayer source reconstruction framework. The study did not involve treatment groups, interventions, or group assignment based on participant characteristics. Comparisons were performed across source models, simulation conditions, and cortical laminar families rather than between participant groups. |
| Blinding | Blinding was not relevant to this study. The analyses were performed on previously collected MEG datasets and did not involve allocation of participants to experimental or treatment groups. Laminar inference, simulations, and model comparisons were conducted using predefined computational pipelines and objective quantitative criteria. Consequently, there were no group assignments from which investigators could be blinded during data collection or analysis. |

# Reporting for specific materials, systems and methods

We require information from authors about some types of materials, experimental systems and methods used in many studies. Here, indicate whether each material, system or method listed is relevant to your study. If you are not sure if a list item applies to your research, read the appropriate section before selecting a response.

## Materials & experimental systems

| n/a | Involved in the study | |
|---|---|---|
| ☒ | ☐ | Antibodies |
| ☒ | ☐ | Eukaryotic cell lines |
| ☒ | ☐ | Palaeontology and archaeology |
| ☒ | ☐ | Animals and other organisms |
| ☒ | ☐ | Clinical data |
| ☒ | ☐ | Dual use research of concern |
| ☒ | ☐ | Plants |

## Methods

| n/a | Involved in the study | |
|---|---|---|
| ☒ | ☐ | ChIP-seq |
| ☒ | ☐ | Flow cytometry |
| ☐ | ☒ | MRI-based neuroimaging |

# Plants

| | |
|---|---|
| Seed stocks | *Report on the source of all seed stocks or other plant material used. If applicable, state the seed stock centre and catalogue number. If plant specimens were collected from the field, describe the collection location, date and sampling procedures.* |
| Novel plant genotypes | *Describe the methods by which all novel plant genotypes were produced. This includes those generated by transgenic approaches, gene editing, chemical/radiation-based mutagenesis and hybridization. For transgenic lines, describe the transformation method, the number of independent lines analyzed and the generation upon which experiments were performed. For gene-edited lines, describe the editor used, the endogenous sequence targeted for editing, the targeting guide RNA sequence (if applicable) and how the editor was applied.* |
| Authentication | *Describe any authentication procedures for each seed stock used or novel genotype generated. Describe any experiments used to assess the effect of a mutation and, where applicable, how potential secondary effects (e.g. second site T-DNA insertions, mosiacism, off-target gene editing) were examined.* |

# Magnetic resonance imaging

## Experimental design

| | |
|---|---|
| Design type | NA |
| Design specifications | NA |

Behavioral performance measures | None

## Acquisition

Imaging type(s): structural

Field strength: 3T

Sequence & imaging parameters: Structural MRI data were acquired on 3 T Siemens systems using dataset-specific protocols. The visually evoked response dataset was acquired on a Siemens Prisma scanner and included T1-weighted MPRAGE (1 mm isotropic), T2-weighted SPACE (0.8 mm isotropic), and quantitative multi-parameter mapping (MPM) acquisitions (0.8 mm isotropic). The visually cued button-press dataset was acquired on a Siemens Magnetom TIM Trio scanner and included T1-weighted FLASH (1 mm isotropic) and quantitative MPM acquisitions (0.8 mm isotropic). The self-paced button-press dataset was acquired on Siemens Prisma or Magnetom Vida systems and included T1-weighted MPRAGE (0.8 mm isotropic), T2-weighted SPACE (0.8 mm isotropic), and multi-echo FLASH acquisitions (1 mm isotropic). Structural MRI data were used for cortical surface reconstruction and generation of multilayer cortical models.

Area of acquisition: whole brain

Diffusion MRI ☐ Used ☒ Not used

## Preprocessing

Preprocessing software: Structural MRI preprocessing was performed using FreeSurfer v6.0.0 and v6.0.1. Processing included brain extraction, tissue segmentation, cortical surface reconstruction, and generation of pial and white/grey matter boundary surfaces using the FreeSurfer recon-all pipeline and dataset-specific procedures based on quantitative multi-parameter mapping (MPM) data where available. Intermediate cortical surfaces were generated using FreeSurfer's equidistant surface generation procedure. The laMEG toolbox (v0.0.7) was subsequently used to downsample the cortical surfaces and construct multilayer cortical models for source reconstruction. No spatial smoothing was applied to the structural MRI data.

Normalization: Structural MRI data were not spatially normalized to a common template for source reconstruction or laminar inference. All cortical surface reconstruction, multilayer surface generation, forward modeling, and source reconstruction were performed in each participant's native anatomical space. For visualization purposes only, selected source locations were projected onto the FreeSurfer fsaverage surface using standard surface-based registration procedures.

Normalization template: Data were not normalized to a common template space. All analyses were performed in each participant's native anatomical space.

Noise and artifact removal: Not applicable. Only structural MRI data were analyzed. No motion-parameter, tissue-signal, heart-rate, or respiration regressors were used. MRI preprocessing was limited to anatomical reconstruction and, where applicable, B1/B0 correction for quantitative MPM images.

Volume censoring: Not applicable. The study used structural MRI acquisitions for anatomical reconstruction and did not involve functional MRI time-series data. Consequently, no volume censoring, scrubbing, or exclusion of individual image volumes was performed.

## Statistical modeling & inference

Model type and settings: The primary analyses used Bayesian model comparison rather than mass-univariate hypothesis testing. For each cortical depth model, source reconstruction was performed using Multiple Sparse Priors (MSP) and model evidence was quantified using variational free energy. Laminar inference was based on comparisons of model evidence across depth-specific source models. Group-level inference was performed using hierarchical random-effects Bayesian model comparison implemented in the Variational Bayesian Analysis (VBA) toolbox, treating participant as a random effect. Cortical layers were grouped into supragranular, granular, and infragranular families, and protected exceedance probabilities (PEPs) were computed at each time point to assess the probability that one laminar family was more likely than the others. Simulation analyses quantified reconstruction performance using laminar reconstruction error, reconstruction bias, and diagonal dominance of model-evidence matrices.

Effect(s) tested: The study evaluated whether multilayer MEG source reconstruction could distinguish activity arising from different cortical depths. Simulation analyses tested the effects of signal-to-noise ratio, co-registration error, cortical column orientation, source extent, noise structure, forward-model mismatch, source-space mismatch, and concurrent sources on laminar inference accuracy. Empirical analyses examined laminar activation dynamics associated with visually evoked fields (VEFs), motor fields (MFs), and motor-evoked field I (MEFI) responses. Statistical inference was performed using Bayesian model comparison across depth-specific source models and across supragranular, granular, and infragranular laminar families. No ANOVA, factorial design, or conventional condition-based hypothesis testing was performed.

Specify type of analysis: ☐ Whole brain ☒ ROI-based ☐ Both

| | |
|---|---|
| Anatomical location(s) | Anatomical locations were defined using participant-specific cortical surface reconstructions generated with FreeSurfer. For the visual dataset, localization was constrained to primary visual cortex (Brodmann area 17) using FreeSurfer's BA_exvivo atlas. For the sensorimotor datasets, localization was constrained to the left precentral gyrus (motor field, MF) and left postcentral gyrus (motor-evoked field I, MEFI) using the FreeSurfer Desikan–Killiany cortical parcellation. Within each anatomically defined region, source localization was performed using an empirical Bayesian beamformer (EBB) within component-specific time windows. A single source vertex was then selected based on the peak EBB response and additional anatomical and biophysical criteria identified in the simulations, including cortical thickness, cortical column orientation, and lead-field separability across cortical depths. Cortical depth surfaces were subsequently mapped to cytoarchitectonic laminae using the BigBrain atlas. |
| Statistic type for inference<br>(See Eklund et al. 2016) | Not applicable. The study did not use voxel-wise or cluster-wise statistical inference. Statistical inference was performed using hierarchical random-effects Bayesian model comparison across participants. Protected exceedance probabilities (PEPs) were computed for laminar model families at each time point using the Variational Bayesian Analysis (VBA) framework. No cluster-forming thresholds, cluster-extent criteria, or voxel-wise significance tests were used. |
| Correction | No frequentist multiple-comparison correction (e.g., FWE, FDR, permutation, or cluster-based correction) was applied. Statistical inference was performed using hierarchical Bayesian model comparison implemented in the Variational Bayesian Analysis (VBA) framework. Group-level inference was based on protected exceedance probabilities (PEPs), which incorporate the Bayesian Omnibus Risk (BOR) to account for the possibility that competing model families are equally likely. Because inference was conducted within a Bayesian model-selection framework rather than through repeated null-hypothesis significance tests, conventional multiple-comparison corrections were not required. |

## Models & analysis

| n/a | Involved in the study |
|---|---|
| ☒ | ☐ Functional and/or effective connectivity |
| ☒ | ☐ Graph analysis |
| ☒ | ☐ Multivariate modeling or predictive analysis |
