# Supplementary material for: Multilayer MEG source modelling enables depth-resolved laminar inference in humans
Source: Nat Commun. 2026 Sep 30;17:10128. doi: 10.1038/s41467-026-77782-9 (PMC13627721; doi:10.1038/s41467-026-77782-9)
Supplement: Supplementary file 3 [file 41467_2026_77782_MOESM3_ESM.pdf]

nature portfolio

Peer Review File

Multilayer MEG source modelling enables depth-resolved laminar inference in humans

Corresponding Author: Dr James Bonaiuto

This file contains all reviewer reports in order by version, followed by all author rebuttals in order by version.

Version 0:

Reviewer comments:

Reviewer #1

(Remarks to the Author)
The manuscript by Szul et al. presents a multilayer high-precision MEG (hpMEG) framework aimed at moving beyond deep-versus-superficial inference toward depth-resolved localization of cortical activities. This is a technically ambitious and sophisticated study that represents a meaningful advance in non-invasive human neuroimaging. However, several concerns regarding the validation and methodology need to be addressed before concluding that MEG can support robust and genuine laminar inference.

Major comments:
(1) The event-related lamina-specific relative model evidence (ΔF) at the peak latency varies monotonically with laminar depth (Figures 8/9/S6/S7). This pattern is not fully convincing, as such monotonicity could arise from systematic biases in the forward model, signal-to-noise gradients across cortical depth, or depth-dependent regularization, rather than reflecting true layer-specific neural activity. The claim of genuine laminar sensitivity would be more compelling if the authors validated their approach using paradigms known to selectively engage intermediate layers.

(2) In classic event-related potential/field (ERP/ERF) analysis, peak latencies within critical time windows often exhibit significant inter-subject variability. Considering that some datasets used in this study contain a vast number of trials per participant (e.g., ~1822 trials per participant in the visually cued button-press dataset), the authors should present individual-level results and evaluate the consistency of the laminar effects across the cohort.

(3) Page 14, Lines 440-456: The authors acknowledge the limitations of current multilayer laminar MEG approaches, particularly regarding the difficulty of resolving two simultaneously active sources. Given that real cortical processing is inherently distributed and overlapping, the authors should provide more detail on how data processing choices, such as the selection of sliding time windows and the use of temporally extended signal space separation (tSSS), might conflate mixed feedforward/feedback or sensorimotor activities.

Minor Comments:
(1) Page 19, Lines 544-545: The authors states “vertex selection was used to maximize laminar discriminability rather than to identify biologically privileged cortical locations”, which reflects a purely data-driven strategy that may substantially inflate ΔF. This raises concerns about potential selection bias in the reported laminar effects. It would therefore be important to clarify why a biologically defined cortical ROI was not used instead.

(2) The authors should clarify the rationale for using different preprocessing pipelines across the three MEG datasets. Although all data were acquired using a 275-channel CTF system, tSSS was applied to the self-paced button-press dataset, SSS to the visually cued dataset, and no spatial filtering to the visually evoked response dataset. Furthermore, the application of ICA and the autoreject pipeline appears inconsistent across datasets. Standardizing the workflow, or providing a robust justification for these discrepancies, is encouraged for ensuring the transparency and reproducibility of the work.

(3) Cortical activity typically induces (de)synchronization in specific frequency bands. The authors are encouraged to provide analysis on beta-band power fluctuations across layers during the button-press tasks. Ideally, a frequency-resolved analysis of feedforward and feedback interactions between layers would significantly strengthen the findings.

(4) There is considerable variance in the number of trials per participant across the empirical datasets. The authors should discuss whether ~200 trials are sufficient for reliable laminar inference of visually evoked fields. Additionally, a comprehensive evaluation of the SNR for all three datasets should be provided.

(Remarks on code availability)
The code is working well, and I have not find problems with the code.

Reviewer #2

(Remarks to the Author)
Authors present a multilayer MEG source modeling approach that is designed to reconstruct sources in the cortex with laminar precision. The approach builds on earlier work by some of the same authors that aimed to disambiguate source contributions from superficial and deep cortical layers. Authors have conducted detailed simulations to characterize the accuracy of laminar source reconstruction and its dependence on SNR, coregistration error and cortical column orientation. In addition, analysis results are presented for evoked fields in visual, motor and somatosensory cortex. The results indicate the feasibility of multilayer MEG source modeling under favorable circumstances.
The topic of this study is highly relevant. Reliable and robust disambiguation of contributions from specific cortical layers to MEG signals would greatly advance the impact and informativeness of MEG research. Indeed, authors perform sophisticated and largely convincing analysis and aim to provide evidence regarding the feasibility of multilayer MEG source modeling. Still, accuracy depends on a large number of factors some of which are difficult to control (and not all of them are actually discussed). So while I am overall positive in my assessment of this study I have some major concerns that need to be addressed. My main concern is that I don't see sufficient evidence to support the claim that depth-resolved inference across all six cortical laminae is possible.

Some of the same authors have worked and published on this approach for a number of years. In several places throughout the manuscript authors basically confirm previous results (e.g. regarding minimum SNR or coregistration error). It would be helpful to state clearly in what ways the current study goes beyond the previous work.

One obvious novel contribution is the extension to multilayser reconstruction. However, I am not entirely convinced to what extent that is really possible. The simulations demonstrate compelling evidence but under artificial conditions (more on that later). The analysis of real data is convincing regarding the differentiation of supra- and infragranular layers. But without knowing the ground truth or having matching invasive recordings it is impossible to validate reconstruction accuracy of individual laminae.

Therefore, I feel that the title (.. ‚enable'...) and some statements in abstract (‚we ... show that laminar precision is achievable') and main text are too strong. What exactly is the evidence to support that claim? (Especially the inference across all six layers)

The strongest evidence regarding the ability to resolve individual cortical laminae comes from the simulations.
However, simulation results can not be directly extrapolated to real data for the following reasons:
a. Real noise is more complex than the simulated white noise.
b. Their simulations do not account for errors in the forward model. As far as I can tell, this point is not even discussed. They use a relatively simple single-shell forward model. Since it is used in the simulation for forward and inverse modeling it does not introduce errors. However, in real data, multilayer results will most likely strongly depend on the accuracy of the forward model. More realistic forward models (like multi-compartment FEM models) might be needed. This is a critical point since their inference is based on leadfield differences between layers and the leadfield computation is based on the volume conductor model (single-shell, BEM, FEM).
c. Simulations are based on single sources (with one exception) and all conclusions regarding minimal SNR and coregistration accuracy are entirely based on single sources. This is a scenario that does not exist in the brain! Therefore, in real data, multilayer results might be affected by nearby sources.
All these points should at least be discussed.

Authors have done a great job with pushing the approach forward through rigorous characterization of their method and putting together a sophisticated analytical and statistical pipeline. However, uncertainties remain for practical use.
In their approach authors capitalize on the minute differences of leadfields across laminae. Still, assigning the location of sources that are represented in noisy recordings to individual laminae is very challenging. In real data, inaccuracies can arise from errors in the forward model and errors in the estimation of cortical column orientation (and these errors are unknown to the researcher). In addition, authors show the effect of patch size on multilayer reconstruction accuracy. That alone can lead to a bias and wrong reconstruction since real patch size is unknown. Also, there is the trade-off between time resolution and SNR for evidence profiles when choosing the temporal window length.
Given all these limitations I would encourage authors to rephrase some of their strong statements (title, abstract and main text) especially pertaining to the inference across six laminae. One example is:
Line 390 „Accurate laminar inference therefore requires a coregistration error of 2 mm or less, though a coarse deep-versus-superficial distinction may be possible in some locations at errors up to 5 mm, provided there is sufficient SNR." Again, this is for the case of white noise, a single source and no error in the forward model and does probably not hold for real data.

Still, my overall assessment of the manuscript is positive. Group statistics (as used here) can partly protect against some

unsystematic errors. Maybe it is even possible to use strong sensory stimuli to generate high-SNR signals as a quality control or reference data (or for some kind of calibration) to gauge individual reconstruction accuracy (similar to a localizer in fMRI) in the same session as your paradigm of interest.

(Remarks on code availability)

Reviewer #3

(Remarks to the Author)
In this work, Szul et al. extend previous work of the group by combining high-precision MEG with fine-grained source models and Bayesian model comparison, moving from the differentiation of deep and superficial cortical sources to depth-resolved inference across all six cortical laminae.

Laminar inference of non-invasive electrophysiology in humans is of high relevance, and moving beyond the deep-superficial dichotomy to full cortical depth resolution is an important step forward in investigating lamina-specific neural dynamics in human cognition. The authors conduct extensive simulations to support the validity of their approach, incorporate post mortem atlases of laminar widths for laminar boundary estimates, and importantly demonstrate the method's applicability to empirical data by reconstructing laminar activity profiles from visual and motor event-related fields, with convincing results. Remaining challenges, such as differentiating multiple sources at different cortical depths, misestimation of patch sizes, and the restriction to a single pre-selected cortical patch location, are clearly delineated in the manuscript.

The present study should be of interest to cognitive and computational neuroscientists seeking to understand cortical computations across layers, as well as clinicians working on diseases with laminar vulnerabilities, such as Parkinson's and Huntington's disease.

General comments

While the manuscript is very clear and well written, the methods descriptions could be improved for clarity, as detailed below.

The experimental findings should be discussed in light of the patch size sensitivity and the limitations of laminar inference for multiple simultaneous sources demonstrated in the simulations. Were different patch sizes tested? Conceptually, I wonder how one would select a patch size for sources known to extend across several vertices?

In general, I would have found more subtitles in the figures helpful. Having to constantly refer back to the legend to read a figure can be tedious. This is only a suggestion.

Specific comments

Lines 150f: Might operationalizing the “error level” as the maximum fiducial displacement lead to a significant overestimation of co-registration error?

Lines 218ff: The definitions of reconstruction error and bias could be clearer: these appear to be averaged values, but it is not immediately obvious from the methods section what is averaged across (locations, depths, etc.) or normalized to what. Some of these details appear later in the results section but should be moved to the methods section for better readability. Additionally, an explanatory sentence addressing why reconstruction errors and biases are observed even though peak relative model evidences all lie on the diagonal (Figures 1f, g, and h) would be helpful.

Lines 251ff: For the analysis of how anatomical features determine the accuracy of laminar inference, I would have expected a mediation analysis, given that we know that dipole orientation relative to the scalp, scalp-to-cortex distance and cortical thickness all impact the leadfield difference. Please comment.

Lines 260ff: “These features were then related to reconstruction error using kernel density estimation and receiver operating characteristic (ROC) analysis to identify anatomical thresholds predictive of accurate inference.” I found this confusing. Earlier in the text (line 252ff), we learn that: “For each vertex, we computed absolute reconstruction error in millimeters. We then analyzed the relationship between reconstruction error and several anatomical properties.” From this, I would have expected reconstruction error to be a continuous variable but later sections of the manuscript suggest it is treated as a binarized measure - no reconstruction error vs. any reconstruction error. This should be made explicit in the manuscript.
How does this reconcile with the four groups displayed in the marginal histograms of Fig. 7d,f,h? How are those defined?

Lines 206f: “Because each time window is evaluated independently in this framework, a single 50 ms window was sufficient for our analysis”. I am not sure I understand this sentence. A single time window in opposition to what?

Lines 227ff: “Effect sizes were computed using Cohen’s d, defined as the difference between observed and shuffled means divided by the standard deviation of the shuffled distribution. We conducted bootstrap resampling (5,000 iterations), drawing random subsets of the data to compute 95% confidence intervals for error and bias.”

Is calling this Cohen's d appropriate? The definition of means differs between the observed mean and the shuffled mean and standard deviations are not pooled. Perhaps simply omit, i.e., "Effect sizes were defined as the difference". Please define random subsets.

Lines 232f: "...the ratio of diagonal values to total values in the free energy matrix.."

Do you mean the ratio of mean diagonal and off-diagonal values?

Lines 339ff: "However, the probability density of surfaces with the maximal free energy
demonstrates that in most locations, the peak free energy corresponded to either the most superficial or the deepest surface model."

Out of curiosity: do you have an explanation for why this is the case? Why the most superficial or deepest surfaces?

Lines 437f: "The increased variability in middle laminae suggests that reconstruction accuracy is not uniform across the cortex, requiring inference strategies that consider regional differences in laminar architecture."

Is this not simply due to the fact that middle layers lie in the middle, while more superficial and deep sources are naturally constrained to have less variability, as they can only be biased in one direction? (There is no deeper layer than the deepest surface.)

Lines 513ff: How is the optimal threshold defined?

Concerning the empirical results: it is commonly assumed that differences in log model evidence should exceed 3 to be considered significant, yet in the present work, statistics are performed on the (non-significant) model evidences. Could you please explain this to the reader?

Figures

- Figure 1c is hard to read. I found Figure 1f confusing, as it does not seem to match the later depictions of "Mean model evidence (free energy, ΔF) matrices (row-normalized to the worst model" (legend of Figure 2). Figure 1f is clearly not row-normalized and uses percentage values instead of row-normalized delta Fs. In addition, I would suggest describing the methodological details in the main manuscript as well, not only in the figure legend.

- The colorbar legend in Figure 4 indicates that thickness is displayed in mm, but the text suggests that proportional thickness is shown. Please clarify. If thickness is indeed displayed in mm, please comment on the cortical thickness across layers, which would then appear to be too small, even for ex vivo data.

- Fig. 4i, 5c-h, 8 and 9: confidence intervals are missing.

- Fig. 7: d,f,h: I think the y-axis label should read "Leadfield RMS difference" not "Leadfield RMSE"? Please mention the optimal threshold in the legend.

Supplementary material

- The shared preprocessing and analysis framework for the empirical data currently leads to substantial duplication of the methodological descriptions. This would be much easier to read (and differences between pipelines easier to spot) if later method descriptions referred back to earlier text and simply highlighted the differences. For example, there is extensive overlap between the last two paragraphs on page 3 and the first two on page 5. The CTF MEG system is referenced three times, each time with a different level of detail.

- The exclusion of 8 subjects with insufficient SNR at the response peak of the visually evoked response dataset should be moved from the supplementary material to the main manuscript.

- The high similarity of the responses in the two motor tasks seems overstated. I do not object to pooling the data, but would suggest toning down the claim of high replicability.

(Remarks on code availability)

Version 1:

Reviewer comments:

Reviewer #1

(Remarks to the Author)
The revised manuscript presents a robust multilayer MEG source reconstruction framework designed for depth resolved laminar inference. The revision has adequately addressed the methodological and interpretative concerns raised during the initial review. The integration of systematic simulations alongside empirical validations across three independent datasets supports the reliability of the proposed framework. The transparent discussion of limitations regarding simultaneous source separation and forward model accuracy ensures appropriate boundary conditions for the findings. The manuscript is structurally sound, scientifically rigorous, and suitable for acceptance in its present form.
Looking ahead, extending this multilayer MEG source reconstruction framework to higher order complex cognitive processes, such as working memory, represents a highly valuable scientific trajectory. Furthermore, this technique holds significant potential for investigating predictive coding in psychosis. Current computational psychiatry holds that the core pathophysiology of conditions like schizophrenia involves dysregulation in the laminar specific routing of prediction error signals and precision weighting across cortical hierarchies. A noninvasive, time-resolved, depth-resolved laminar inference technique can provide the necessary empirical means to directly quantify these abnormal communications at specific cortical layers, thereby offering crucial neurophysiological evidence for the predictive coding dysfunction hypothesis.
Minor comment
Lines 1082-1084: The data repository link provided within the revision in the “Data availability” section (https://doi.org/10.5281/zenodo.20721185) is currently inaccessible (DOI not found). To facilitate a thorough evaluation and uphold research transparency, I encourage the authors to provide a secure reviewer access link for the preprocessed data.

(Remarks on code availability)
The code is well-organized and reproducible. The repository provides a clear README with straightforward instructions, and it is easy to install and run for reproducibility.

Reviewer #2

(Remarks to the Author)
Authors have addressed my concerns and I am happy to recommend publication.
The revision is very comprehensive and includes a lot of new computations and analysis.
A small minor comment: In the revision, when discussing possible effects of additional sources, colored noise or different forward models, they conclude on several occasions:
"Nevertheless, the overall structure of the results remained similar to the primary simulations, with reconstruction accuracy improving monotonically with increasing SNR and decreasing co-registration error (Supplementary Fig. 9 and 13)."
A monotonic improvement of any performance measure with increasing SNR and decreasing co-registration is rather trivial and not very interesting. It is more interesting to conclude under what conditions accurate laminar inference is still possible.
But overall, I am happy with this manuscript since all the data is clearly presented in the main text and Suppl Mat.

(Remarks on code availability)

Reviewer #3

(Remarks to the Author)
I thank the authors for their thoughtful and thorough revision of the manuscript. The methods section has been substantially clarified and now provides a much clearer description of the experimental design and analytical approach. The extensive supplementary analyses the authors have added strengthen the robustness of their conclusions. I have no further concerns and am happy to recommend this manuscript for publication.

(Remarks on code availability)

Open Access This Peer Review File is licensed under a Creative Commons Attribution 4.0 International License, which permits use, sharing, adaptation, distribution and reproduction in any medium or format, as long as you give appropriate credit to the original author(s) and the source, provide a link to the Creative Commons license, and indicate if changes were made.
In cases where reviewers are anonymous, credit should be given to 'Anonymous Referee' and the source.
The images or other third party material in this Peer Review File are included in the article's Creative Commons license, unless indicated otherwise in a credit line to the material. If material is not included in the article's Creative Commons license and your intended use is not permitted by statutory regulation or exceeds the permitted use, you will need to obtain permission directly from the copyright holder.
To view a copy of this license, visit https://creativecommons.org/licenses/by/4.0/

Reviewer #1

The manuscript by Szul et al. presents a multilayer high-precision MEG (hpMEG) framework aimed at moving beyond deep-versus-superficial inference toward depth-resolved localization of cortical activities. This is a technically ambitious and sophisticated study that represents a meaningful advance in non-invasive human neuroimaging. However, several concerns regarding the validation and methodology need to be addressed before concluding that MEG can support robust and genuine laminar inference.

Major comments:

1. The event-related lamina-specific relative model evidence (ΔF) at the peak latency varies monotonically with laminar depth (Figures 8/9/S6/S7). This pattern is not fully convincing, as such monotonicity could arise from systematic biases in the forward model, signal-to-noise gradients across cortical depth, or depth-dependent regularization, rather than reflecting true layer-specific neural activity. The claim of genuine laminar sensitivity would be more compelling if the authors validated their approach using paradigms known to selectively engage intermediate layers.

   We agree that a monotonic variation in peak model evidence across laminar depth could, in principle, arise from biases in the forward model, depth-dependent SNR differences, or regularization effects, rather than reflecting true laminar specificity. However, our interpretation of laminar sensitivity does not rely on the static depth profile at the peak latency. Instead, it is based on the temporal evolution of model evidence, which reveals systematic and physiologically interpretable changes in the dominant laminar model over time. In both visual and sensorimotor datasets, we observe clear transitions in the laminar profile (e.g., early granular or infragranular dominance followed by supragranular activity), which would not be expected under a fixed bias such as depth-dependent SNR or forward model asymmetries. Such biases would be expected to produce a consistent preference for specific depths across time, rather than the observed dynamic shifts in model evidence. Moreover, the layer-specific source models are matched in complexity: corresponding cortical surfaces contain identical numbers of vertices due to one-to-one downsampling across depth, and dipole orientations are constrained using link vectors between corresponding vertices. Consequently, differences in model evidence between layers reflect differences in source depth and associated lead fields, rather than differences in model complexity or orientation priors (lines 1048-1062):

   In order to interpret the cortical depth surfaces in terms of laminae, we mapped the BigBrain atlas to each subject's anatomy, computed proportional laminae boundaries from the atlas, scaled these boundaries by the local cortical thickness, and assigned each of the 11 cortical surface layers to a corresponding cytoarchitectonic lamina. Free energy values were then averaged within laminae. The resulting model evidence was then compared in two ways: i) by computing model evidence relative to the worst model (ΔF) at each time point, and ii) using Bayesian model selection to identify the most probable family of models at the group level at each time point. Because all cortical

depth surfaces were generated by jointly downsampling the multilayer mesh while preserving one-to-one vertex correspondence across depth, each layer-specific source model contained the same number of dipoles and free parameters. Furthermore, dipole orientations were constrained using link vectors connecting corresponding vertices across surfaces, ensuring identical orientation priors across layer models. Consequently, differences in model evidence between layers reflect differences in source depth rather than differences in model complexity, dipole orientation, or source-space dimensionality.

In addition, our simulations explicitly demonstrate that the model comparison framework is capable of correctly recovering the true laminar source under realistic SNR and co-registration conditions. These results suggest that while depth-dependent biases may influence absolute model evidence values, they do not preclude accurate inference of relative laminar contributions.

We also note that orderly depth-dependent gradients are not unique to hpMEG, but are commonly observed in laminar fMRI studies, where they are generally interpreted in the context of structured cortical microcircuit organization rather than solely as methodological bias (Lawrence et al., 2019; Uludag & Havlicek, 2021; Qin et al., 2026). While laminar fMRI and MEG are sensitive to different physiological signals and are subject to distinct sources of bias, the presence of systematic depth-dependent structure across modalities supports the plausibility of temporally evolving laminar profiles in non-invasive recordings.

We agree that paradigms selectively engaging intermediate layers would provide a valuable additional validation. However, even in well-characterized sensory paradigms, thalamocortical input to layer IV is rapidly followed by activation of supra- and infragranular layers, making isolated engagement of intermediate layers difficult to achieve. We have revised the manuscript to clarify that our conclusions are based on the temporal dynamics of laminar model evidence, rather than solely on peak depth profiles, and to explicitly acknowledge the potential influence of depth-dependent biases (lines 530-557):

Accurate laminar inference ultimately depends on the fidelity of the forward model. A potential concern is that monotonic variations in peak model evidence across cortical depth could reflect depth-dependent biases in the forward model, signal-to-noise gradients, or regularization effects rather than true laminar specificity. However, our interpretation does not rely on static depth profiles at a single latency, but on the temporal evolution of laminar model evidence, which shows systematic shifts in dominant laminar contributions over time. Such dynamic transitions are not readily explained by fixed biases. Moreover, because the layer-specific source models are matched in complexity and orientation priors through vertex correspondence and link vector constraints, differences in model evidence primarily reflect depth-dependent differences in the forward model rather than differences in model dimensionality. Nonetheless, resolving activity from intermediate laminae remains challenging, particularly under lower SNR conditions. The simulations further demonstrated that lead field separability between superficial and deep cortical layers is the strongest anatomical

predictor of laminar inference accuracy, emphasizing the importance of accurate forward modeling. To assess robustness to forward-model assumptions, we performed additional simulations incorporating source-space mismatch, forward-model mismatch, and more realistic noise conditions. While source-space mismatch produced only modest reductions in reconstruction accuracy, simulations generated using a BEM forward model and reconstructed using the single-shell model employed throughout this study showed larger performance reductions, particularly at lower SNRs and higher co-registration errors. Nevertheless, the qualitative dependence of reconstruction accuracy on SNR and co-registration precision remained unchanged across all robustness analyses. These findings suggest that the principal conclusions are reasonably robust to moderate model mismatch while also highlighting forward-model accuracy as a primary determinant of achievable laminar precision in empirical data. Future work could improve laminar inference precision by employing more sophisticated forward models, such as BEM or finite element models (FEMs). Targeted sensory paradigms that preferentially engage specific cortical laminae may also provide an important avenue for further validation of laminar specificity.

2. In classic event-related potential/field (ERP/ERF) analysis, peak latencies within critical time windows often exhibit significant inter-subject variability. Considering that some datasets used in this study contain a vast number of trials per participant (e.g., ~1822 trials per participant in the visually cued button-press dataset), the authors should present individual-level results and evaluate the consistency of the laminar effects across the cohort.

   We thank the reviewer for this comment. We agree that inter-subject variability in the timing and magnitude of event-related responses is an important consideration. However, the primary statistical analyses in this study were performed using random-effects Bayesian model selection (RFX-BMS), which explicitly evaluates the consistency of model evidence across participants rather than relying on group-averaged responses. Specifically, at each time point, family-wise Bayesian model comparison was performed on the subject-level free-energy values, treating model identity as a random variable across participants. Protected exceedance probabilities therefore quantify the probability that a given laminar family is more frequent in the population than competing alternatives while accounting for between-subject variability and uncertainty in model frequencies (Stephan et al., 2009; Rigoux et al., 2014). Consequently, the reported laminar effects are not driven by a small subset of participants with large free-energy differences, but instead reflect effects that are consistently expressed across the cohort. To clarify this point, we have expanded the description of the statistical framework in the Methods and Results sections and now explicitly state that group-level inference was performed using random-effects Bayesian model comparison on subject-level model evidence rather than on averaged free-energy traces (lines 1069-1081):

   For Bayesian model selection we used the VBA (Variational Bayesian Analysis) toolbox42. The free energy measures of the six laminae were grouped in three families:

supragranular (laminae I-III), granular (lamina IV) and infragranular (laminae V-VI) families. At each time point, family-wise random effects Bayesian model selection was applied to the subject-level free energy values. This approach treats model identity as a random variable across participants and estimates the population frequency of each laminar family, thereby accounting for inter-subject variability in model preference43,44. In the case when the posterior probability that all families are equally frequent in the population was below 0.5, we calculated protected exceedance probabilities (PEPs) to quantify the probability that one laminar family was more frequent in the population than the competing alternatives while accounting for chance-level differences in model frequency. Consequently, group-level inference was based on the consistency of model evidence across participants rather than on averaged free energy values.

We have also tried to make this clear in the Results section (lines 439-444):

To assess consistency across participants, we performed family-wise random-effects Bayesian model selection[42–44], grouping laminae into supragranular (I-III), granular (IV), and infragranular (V-VI) families. Protected exceedance probabilities (PEPs) were computed at each time point to quantify the likelihood that one laminar family dominated over the others while accounting for chance-level equivalence[43,44].

3. Page 14, Lines 440-456: The authors acknowledge the limitations of current multilayer laminar MEG approaches, particularly regarding the difficulty of resolving two simultaneously active sources. Given that real cortical processing is inherently distributed and overlapping, the authors should provide more detail on how data processing choices, such as the selection of sliding time windows and the use of temporally extended signal space separation (tSSS), might conflate mixed feedforward/feedback or sensorimotor activities.

We agree that temporal processing choices could potentially influence laminar inference. The original manuscript included simulations demonstrating that laminar inference remained accurate across a range of sliding-window sizes, but that increasing window length progressively smoothed the temporal profile of model evidence. To assess the practical impact of this effect, we additionally repeated the empirical VEF, MF, and MEF-I analyses using window sizes of 10, 25, and 50 ms (Supplementary Figure 19). These analyses demonstrated that short windows better resolve brief laminar transients, whereas longer windows provide greater sensitivity at the cost of temporal specificity. For example, the transient granular response in the visual ERF was only clearly resolved using a 10 ms window, whereas the characteristic infra-/supragranular dynamics of the motor responses were more robustly detected using 25-50 ms windows. We have therefore expanded the Discussion to explicitly discuss the trade-off between temporal resolution and sensitivity in sliding-window laminar inference (lines 616-633):

Another methodological consideration concerns the temporal window over which model evidence is estimated. In supplementary simulations using different sliding window sizes (15, 25, 50, and 100 ms; Supplementary Fig. 1), we assessed the temporal sensitivity of

laminar model evidence. Across all window lengths, model-based inference consistently recovered the true laminar depth, but the temporal profile of model evidence differed. Narrow windows (e.g., 15 ms) yielded sharper but noisier evidence profiles that collapsed earlier, while wider windows (e.g., 100 ms) integrated more information, resulting in broader and more stable evidence plateaus over time. Consistent with these simulations, empirical analyses repeated using 10, 25, and 50 ms windows (Supplementary Fig. 19) showed that short windows better resolved brief laminar transients, whereas longer windows produced smoother and more temporally extended evidence profiles. For example, the transient granular response in the visual ERF was most clearly detected using a 10 ms window, whereas the characteristic infra-/supragranular dynamics of the MF and MEF-I were more robustly detected using 25-50 ms windows. This trade-off reflects the balance between temporal resolution and sensitivity: short windows enable finer tracking of rapid changes but require high SNR, whereas longer windows are more robust to noise but may obscure transient laminar dynamics. Accordingly, window size should be selected according to the expected temporal scale of the neural process under investigation.

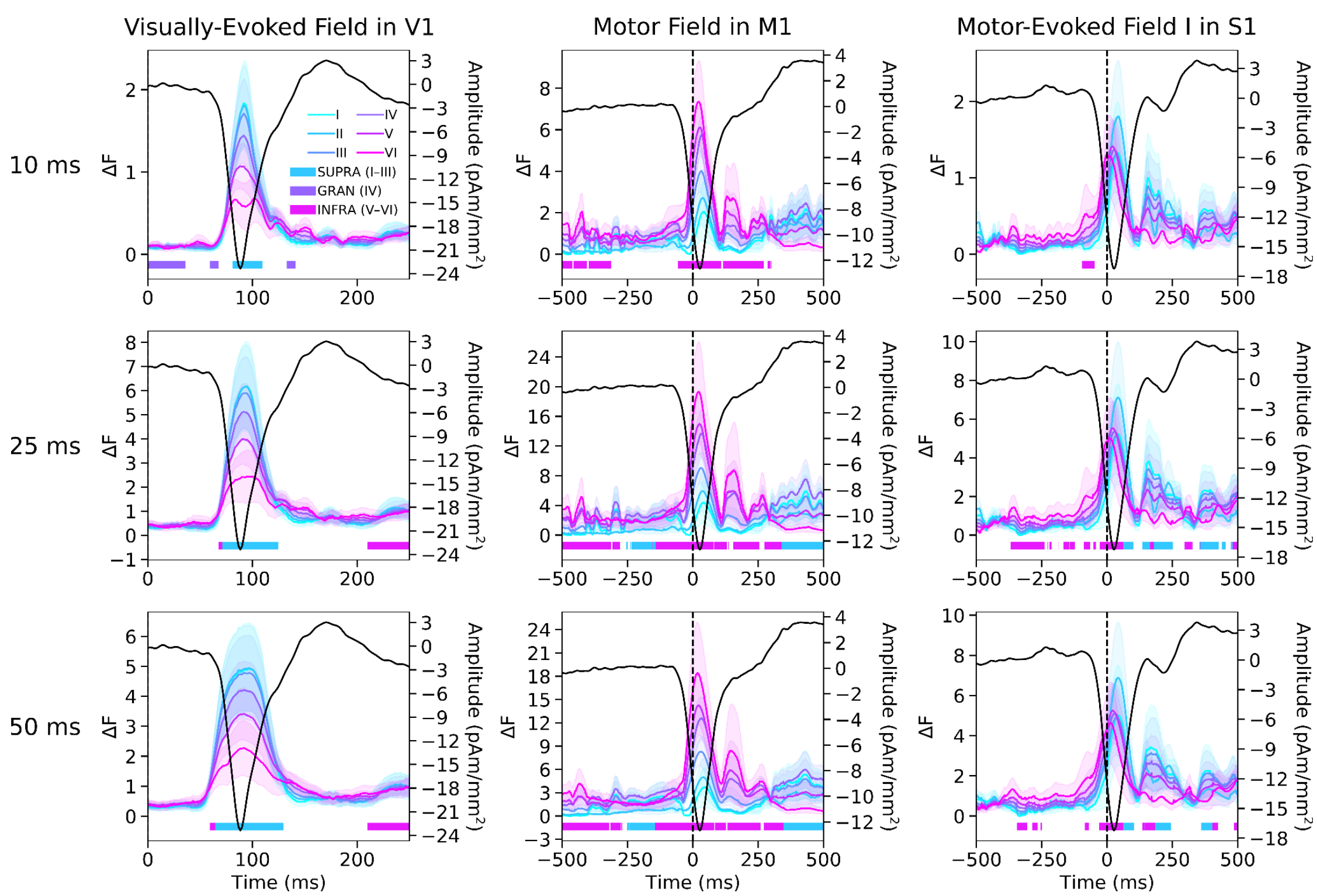

Supplementary Figure 19. **Sensitivity of empirical laminar inference to sliding-window duration.** Sliding-window laminar inference results for the visually evoked field (VEF), motor field (MF), and motor-evoked field I (MEF-I; columns) using window sizes of 10 ms, 25 ms, and 50 ms (rows). Colored traces show lamina-specific relative model evidence (ΔF, relative to the worst model at each time point) averaged across participants, with shaded regions denoting SEM. Dark traces show the corresponding middle-layer source time series. Horizontal color bars indicate, at each time point, the laminar family with the highest protected exceedance probability (PEP), grouping layers into supragranular (laminae I-III), granular (lamina IV), and infragranular (laminae V-VI) families. Short windows provide greater temporal specificity and better resolve

brief laminar transients, whereas longer windows integrate information over time and produce smoother, more temporally extended evidence profiles. In the VEF, the transient granular response is most clearly resolved using a 10 ms window. In contrast, the characteristic infra-/supragranular dynamics of the MF and MEF-I are more robustly detected using 25-50 ms windows. These results demonstrate the trade-off between temporal resolution and sensitivity in sliding-window laminar inference.

With regard to tSSS, we note that the visual dataset was analyzed without tSSS whereas the button-press datasets were preprocessed using tSSS. Despite these differences in preprocessing, the empirical datasets exhibited physiologically plausible and internally consistent laminar dynamics. While tSSS may alter the covariance structure of the sensor data, we did not observe evidence that it qualitatively changed the inferred laminar sequences. Nevertheless, we agree that the influence of preprocessing choices on laminar inference warrants systematic investigation in future work and have added this point to the Discussion (lines 634-641):

More generally, preprocessing choices may also influence the temporal and spatial characteristics of the reconstructed signals. For example, the button-press datasets were preprocessed using temporally extended signal space separation (tSSS)[62], whereas the visual dataset was not. Although tSSS can alter the covariance structure of the sensor data, the broadly consistent physiological patterns recovered across these independently acquired datasets suggest that the principal laminar findings are not driven by this preprocessing step. Nevertheless, the influence of preprocessing approaches, including tSSS and related denoising methods, on laminar inference accuracy remains an important topic for future work.

With regard to concurrent activity, we performed additional simulations including multiple simultaneously active cortical sources in response to comment 2 from reviewer 2. Although reconstruction accuracy was modestly reduced, the principal conclusions regarding SNR and co-registration requirements remained unchanged. These simulations further support the interpretation of multilayer hpMEG as identifying the dominant laminar contribution within a localized cortical region and time window, rather than fully resolving all simultaneously active laminar generators independently (lines 322-328):

Second, we evaluated robustness to concurrent activity by introducing three additional interfering sources at random cortical locations. Each interfering source had an independent band-limited temporal profile and an amplitude equal to 10% of the primary source. Although reconstruction accuracy was modestly reduced, reliable laminar inference still emerged between approximately -35 and -20 dB SNR and deteriorated beyond approximately 2-3 mm co-registration error, closely matching the results of the primary simulations (Supplementary Fig. 7 and 11).

We have expanded our treatment of this point in the Discussion (lines 575-594):

Despite the significant improvements offered by our multilayer approach, a key limitation is the inability of current model-based inference methods to resolve two simultaneous sources of equal magnitude at different depths. Additional simulations including three weaker interfering sources at separate cortical locations produced only modest reductions in reconstruction accuracy, suggesting that the framework is reasonably robust to low-level concurrent activity. However, the inability to resolve two simultaneous sources of comparable magnitude at different depths indicates that the current approach should be interpreted as identifying the dominant laminar contribution within a localized cortical region and time window, rather than independently resolving all simultaneously active laminar generators. Although exact temporal alignment and perfectly matched source magnitudes are unlikely in biological systems, this limitation highlights the constraints of relying solely on Bayesian model comparison. One potential solution would be to use sensors capable of measuring higher spatial-frequency components of the magnetic fields, such as optically pumped magnetometers (OPMs), which could increase sensitivity to subtle spatial differences between closely spaced sources60. Future refinements should explore both sensor technology advances and alternative inference strategies, such as Bayesian model averaging (BMA), which assigns probabilistic weights to multiple competing models rather than selecting a single best-fit model61. These combined approaches may enhance the simultaneous resolution of multiple sources, overcoming a fundamental limitation of the current winner-takes-all approach.

Minor Comments:

1. Page 19, Lines 544-545: The authors states "vertex selection was used to maximize laminar discriminability rather than to identify biologically privileged cortical locations", which reflects a purely data-driven strategy that may substantially inflate ΔF. This raises concerns about potential selection bias in the reported laminar effects. It would therefore be important to clarify why a biologically defined cortical ROI was not used instead.

We agree that unconstrained, data-driven vertex selection could introduce bias and inflate model evidence if selection were based on the same quantity used for inference. However, this was not the case in our analysis. Vertex selection was not performed to maximize ΔF or any laminar model comparison metric. Instead, it was based on independent criteria: (i) functional localization using an empirical Bayes beamformer (EBB), and (ii) anatomical and biophysical constraints identified in our simulations (e.g., cortical thickness, dipole orientation, and lead field separability). These criteria determine whether laminar inference is theoretically feasible at a given location and are orthogonal to the laminar model evidence itself.

Importantly, our simulations demonstrate that laminar discriminability varies substantially across the cortex and is strongly constrained by anatomical and forward-model properties. Performing laminar inference at locations that do not meet these conditions would not provide an unbiased estimate, but rather yield uninformative or misleading results due to poor identifiability. In this sense, vertex selection reflects a principled restriction of the analysis to regions where the inverse problem is sufficiently

well-conditioned, analogous to restricting analyses in fMRI to regions with adequate signal quality.

Purely anatomically defined regions may include large areas where laminar discrimination is not feasible. Our approach therefore combines functional localization with anatomically informed constraints to identify regions that are both biologically relevant and suitable for laminar analysis. To address this concern, we have clarified in the manuscript that vertex selection was based on independent anatomical and functional criteria, and not on laminar model evidence (lines 428-434):

Vertex selection was based on independent functional localization and anatomical constraints identified in simulations (e.g., cortical thickness, dipole orientation, and lead field separability), which determine whether laminar inference is feasible at a given location (see Methods). This approach prioritizes regions where the inverse problem is sufficiently well-conditioned for laminar discrimination, rather than applying laminar inference uniformly across anatomically defined regions where identifiability may be poor.

2. The authors should clarify the rationale for using different preprocessing pipelines across the three MEG datasets. Although all data were acquired using a 275-channel CTF system, tSSS was applied to the self-paced button-press dataset, SSS to the visually cued dataset, and no spatial filtering to the visually evoked response dataset. Furthermore, the application of ICA and the autoreject pipeline appears inconsistent across datasets. Standardizing the workflow, or providing a robust justification for these discrepancies, is encouraged for ensuring the transparency and reproducibility of the work.

    We have clarified the preprocessing descriptions throughout the manuscript and corrected a terminology error: the visually cued button-press dataset used temporal Signal Space Separation (tSSS), not standard SSS. The preprocessing differences across datasets were intentional and reflected differences in acquisition conditions and artifact structure rather than arbitrary pipeline choices. The visually evoked response dataset was acquired in a single supine session with minimal head movement and strong eye-tracking-based trial rejection, making movement compensation unnecessary. In contrast, both button-press datasets involved movement-related artifacts and required concatenation across blocks or sessions, motivating the use of tSSS with continuously tracked head-position indicator coils to compensate for slow head drift and align data to a common head coordinate frame. ICA-based artifact correction was used in the motor datasets because of the greater prevalence of movement-, muscle-, and blink-related artifacts associated with button-press behavior, whereas the visually evoked dataset instead relied primarily on strict eye-tracking-based epoch rejection and manual inspection. Autoreject was additionally applied only in the self-paced dataset because the unconstrained timing of self-initiated responses introduced greater trial-to-trial variability and residual artifacts than the visually cued task.

    Importantly, we view the ability to recover qualitatively consistent laminar dynamics across datasets processed with somewhat different preprocessing pipelines as

a strength of the approach. The results suggest that multilayer hpMEG does not depend on a narrowly defined preprocessing workflow, but can be applied across datasets acquired under different experimental and preprocessing conditions, provided that sufficient SNR and co-registration accuracy are achieved. We have revised the Supplementary Materials to make the rationale for these preprocessing choices explicit and to standardize the methodological descriptions across datasets.

Supplementary Material:

Visually cued button-press dataset

…

Because this dataset was acquired in an upright seated position across multiple sessions, preprocessing differed slightly from that used for the visually evoked response dataset. In particular, small between-block and between-session head-position shifts required explicit movement compensation and cross-session realignment, which were not necessary in the visually evoked dataset acquired in a single supine session. In addition, line-noise removal and artifact correction procedures were adapted for the longer continuous recordings and motor-related artifacts characteristic of the button-press task.

…

Self-paced button-press dataset

…

MEG data were recorded using the same CTF system used to record the visually evoked response dataset at 1200 Hz. In a single session, participants completed two blocks of an auditory oddball task followed by two blocks of a multi-tone masker task, described in detail elsewhere[16]. Between trials, participants initiated the next trial by pressing a button with their right index finger on a response box. Analyses in the present study focused exclusively on these self-paced button-press events, which occurred independently of auditory stimulation.

Participants wore individualized head-casts to stabilize head position during MEG acquisition[3] while in the supine position. Unlike the visually cued button-press dataset, the fiducial coils (nasion, LPA, RPA) were taped directly to the participant's skin to track head motion relative to the cast. To recover the coil positions in native MRI space, each participant's head and fiducials were digitized using an Artec Eva handheld 3D scanner. The resulting 3D mesh was cropped to the facial region and co-registered to the MRI-derived scalp surface (from the synthetic FLASH image) using a combination of fiducial-based alignment and iterative closest point (ICP) registration implemented in MNE-Python. The final fiducial coordinates were used for MEG-MRI co-registration in subsequent laminar source reconstruction analyses. Each session comprised 316-332 button presses per participant (M = 328.8).

Preprocessing procedures were exactly the same as those used for the visually cued button-press dataset, with the exception that because the self-paced responses exhibited greater trial-to-trial variability in movement timing and associated artifacts,

autoreject[17] was applied to the session epochs to automatically detect and repair residual artifacts using cross-validated consensus thresholds (consensus = 0-1, n_interpolate = [1, 4, 32]) before averaging over trials.

Localization of the MF and MEFI components of the motor ERF used exactly the same anatomical ROIs (left precentral and postcentral gyri) and time windows used for the visually cued button-press dataset. Two participants were excluded from the MF analysis and one from the MEFI analysis due to having components that localized outside of the hand area of the primary motor or somatosensory cortex, respectively. All preprocessing and laminar analysis code for this dataset can be found at https://github.com/danclab/auditory_laminar.

3. Cortical activity typically induces (de)synchronization in specific frequency bands. The authors are encouraged to provide analysis on beta-band power fluctuations across layers during the button-press tasks. Ideally, a frequency-resolved analysis of feedforward and feedback interactions between layers would significantly strengthen the findings.

We thank the reviewer for this insightful suggestion. We agree that frequency-resolved analyses of laminar dynamics, including beta-band activity and interlaminar interactions, are of considerable interest. However, the present framework is specifically designed for model-based inference on time-resolved evoked responses (ERFs), where signals are phase-locked and can be averaged across trials. Prior work using high-precision MEG has demonstrated lamina-specific frequency-resolved power changes using a two-layer framework (Bonaiuto et al., 2018). In addition, laminar dynamics of beta bursts have been investigated by aligning bursts to their peak and treating them analogously to evoked responses (Bonaiuto et al., 2021). While informative, this approach effectively recasts transient oscillatory events as ERF-like signals and therefore does not constitute a fully frequency-resolved or continuous oscillatory analysis. Extending the present multilayer framework to genuinely frequency-resolved or ongoing oscillatory dynamics would require substantial methodological developments that explicitly account for non-phase-locked activity and transient burst structure, as well as inference procedures capable of resolving interlaminar interactions in the spectral domain. We have now clarified this distinction in the Discussion (lines 642-651) and highlighted this as an important direction for future work:

While the present study focuses on time-resolved laminar inference of evoked responses, an important extension will be to characterize laminar dynamics of oscillatory activity. Previous work has examined laminar aspects of oscillatory activity using two-layer models, including frequency-resolved power analyses[24] and burst-aligned averaging approaches[25]. However, these approaches either rely on steady-state power estimates or recast transient burst events as ERF-like signals, and therefore do not capture fully time-frequency-resolved laminar interactions. Extending the current multilayer framework to such non-phase-locked dynamics will require analyses that

explicitly capture transient and frequency-specific activity, as well as inference methods capable of resolving interlaminar interactions. Developing such approaches represents an important direction for future work.

4. There is considerable variance in the number of trials per participant across the empirical datasets. The authors should discuss whether ~200 trials are sufficient for reliable laminar inference of visually evoked fields. Additionally, a comprehensive evaluation of the SNR for all three datasets should be provided.

This is an important point to consider. To assess the relationship between trial count and signal quality, we quantified sensor-level SNR within the component-specific analysis windows for all empirical datasets and visualized these as a function of the number of trials (Supplementary Fig. 14):

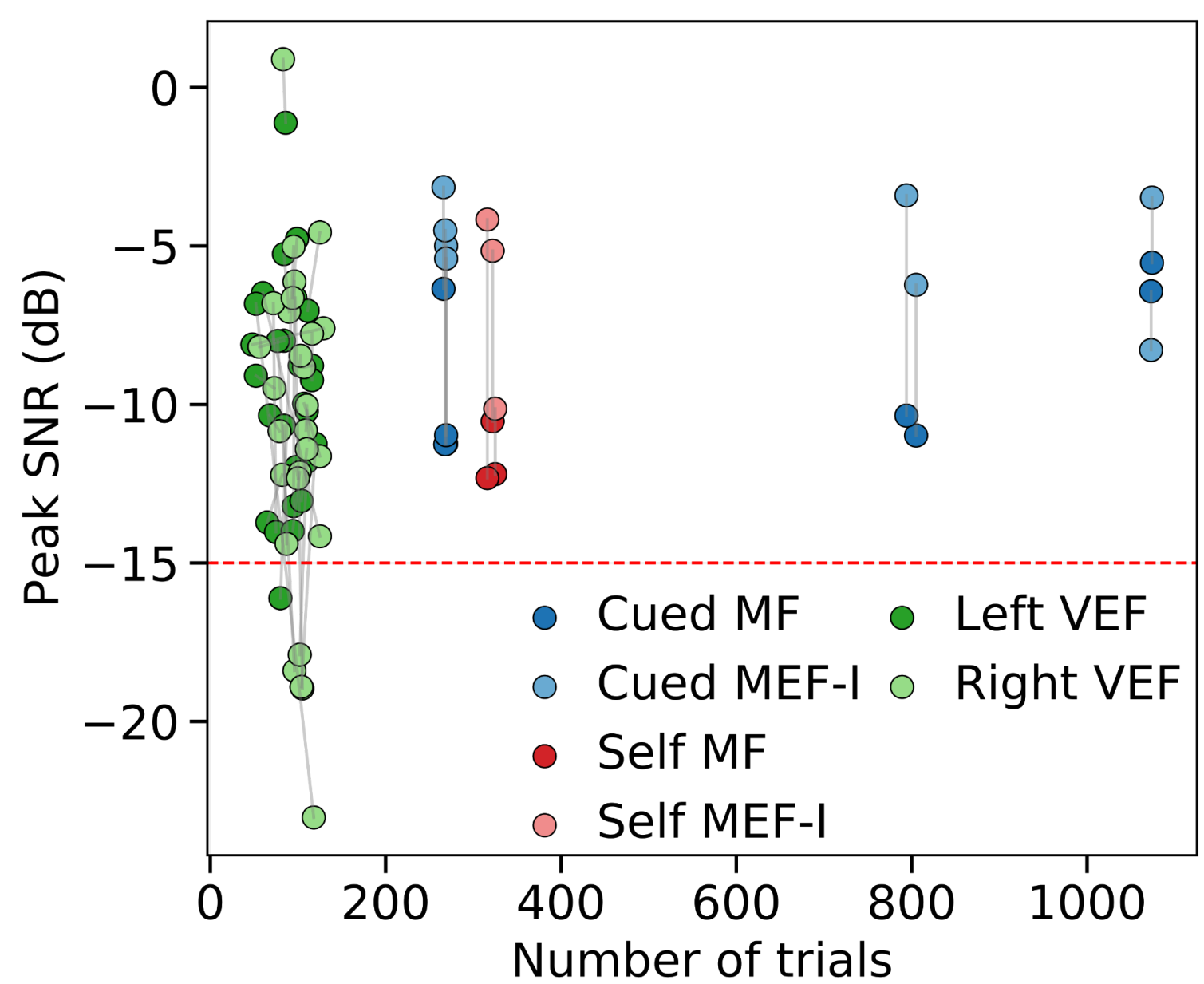

Supplementary Figure 14. **Relationship between trial count and signal-to-noise ratio (SNR) across datasets.** Sensor-level SNR (dB) within component-specific time windows plotted against the total number of trials for each participant and dataset. Data are shown separately for motor field (MF) and motor-evoked field I (MEF-I) components in the visually cued and self-paced button press datasets, and left and right stimulus presentation in the visually evoked response dataset. The gray lines connect datapoints from the same subject. SNR was computed as the ratio of the RMS of the trial-averaged signal to the RMS of trial-by-trial deviations. Considerable variability in SNR is observed across participants, datasets, and components with no simple linear relationship to trial count. The red dashed horizontal line indicates the SNR threshold (-15 dB) used for participant exclusion.

Across datasets, SNR varied substantially, reflecting differences in data quality, component strength, and task structure. Importantly, laminar inference in the present study is performed on trial-averaged evoked responses (ERFs), rather than single-trial data. As a result, the relevant determinant of inference accuracy is the quality of the

averaged response, which depends on SNR rather than trial count per se. We have clarified this point in the Methods section (lines 1017-1025):

All laminar inference analyses were performed on trial-averaged ERFs, rather than on single-trial data. Sensor-level SNR was therefore used as the primary measure of data quality, reflecting the reliability of the averaged response (Supplementary Fig. 14). Sensor-level SNR was computed within the analysis window as the ratio in dB of the RMS of the trial-averaged signal across channels to the RMS of trial-by-trial deviations from that mean, yielding a single SNR time series per participant. Participants whose SNR within this window was < -15 dB were excluded (visually evoked response dataset: *N* = 6; visually cued button press dataset: *N* = 0; self-paced button press dataset: *N* = 0).

Empirical studies have shown that robust ERFs can be achieved with 50-100 trials when appropriate preprocessing and SNR-based quality control are applied (e.g., Huffmeijer et al., 2014; Boudewyn et al., 2018). In line with this, our datasets, including those with ~200 trials, yielded robust ERFs when SNR exceeded the applied inclusion threshold (-15 dB), and participants with insufficient SNR were excluded. Consistent with our simulations, these results indicate that laminar inference is primarily constrained by achievable SNR rather than the absolute number of trials (lines 415-423):

Prior to laminar analysis, we quantified sensor-level signal-to-noise ratio (SNR) within the component-specific analysis windows and examined its relationship to trial count across participants. Across datasets, both trial count and sensor-level SNR varied substantially, with no simple linear relationship between the two (Supplementary Fig. 14). This likely reflects differences in component amplitude, noise structure, and preprocessing efficacy across paradigms and participants. Because laminar inference was performed on trial-averaged ERFs rather than single-trial activity, reconstruction performance depends primarily on the SNR of the averaged response rather than on trial count per se. Participants whose averaged responses exceeded an SNR threshold of -15 dB were retained for laminar analysis.

lines 741-745:

These findings further suggest that there is unlikely to be a universal minimum trial count required for laminar inference. Instead, the achievable precision will depend on the interaction between trial count, component magnitude and variability, preprocessing quality, head stabilization, and recording noise characteristics, all of which determine the effective SNR of the evoked response.

Reviewer #2

Authors present a multilayer MEG source modeling approach that is designed to reconstruct sources in the cortex with laminar precision. The approach builds on earlier work by some of the same authors that aimed to disambiguate source contributions from superficial and deep cortical layers. Authors have conducted detailed simulations to characterize the accuracy of laminar

source reconstruction and its dependence on SNR, coregistration error and cortical column orientation. In addition, analysis results are presented for evoked fields in visual, motor and somatosensory cortex. The results indicate the feasibility of multilayer MEG source modeling under favorable circumstances.
The topic of this study is highly relevant. Reliable and robust disambiguation of contributions from specific cortical layers to MEG signals would greatly advance the impact and informativeness of MEG research. Indeed, authors perform sophisticated and largely convincing analysis and aim to provide evidence regarding the feasibility of multilayer MEG source modeling. Still, accuracy depends on a large number of factors some of which are difficult to control (and not all of them are actually discussed). So while I am overall positive in my assessment of this study I have some major concerns that need to be addressed. My main concern is that I don't see sufficient evidence to support the claim that depth-resolved inference across all six cortical laminae is possible.

1. Some of the same authors have worked and published on this approach for a number of years. In several places throughout the manuscript authors basically confirm previous results (e.g. regarding minimum SNR or coregistration error). It would be helpful to state clearly in what ways the current study goes beyond the previous work. One obvious novel contribution is the extension to multilayser reconstruction. However, I am not entirely convinced to what extent that is really possible. The simulations demonstrate compelling evidence but under artificial conditions (more on that later). The analysis of real data is convincing regarding the differentiation of supra- and infragranular layers. But without knowing the ground truth or having matching invasive recordings it is impossible to validate reconstruction accuracy of individual laminae. Therefore, I feel that the title (.. ‚enable'...) and some statements in abstract (‚we ... show that laminar precision is achievable') and main text are too strong. What exactly is the evidence to support that claim? (Especially the inference across all six layers)

    We agree that the original framing did not sufficiently distinguish between the contributions of the present study and our previous work, and that some statements about "all six laminae" and "laminar precision" were too strong given the distinction between simulation-based feasibility and empirical validation. Previous work from our group established three main points: first, that MEG can distinguish deep from superficial sources under high-SNR and low-coregistration-error conditions (Troebinger et al., 2014; Bonaiuto et al., 2018a); second, that this approach can be applied to frequency-resolved alpha, beta, and gamma activity using a two-layer model (Bonaiuto et al., 2018b; Bonaiuto et al., 2021); and third, that accurate cortical-column orientation estimates improve MEG source inversion (Bonaiuto et al., 2020). The present study goes beyond this work in several ways. Specifically, we: (i) extend the model space from two surfaces to a multilayer cortical source model spanning the cortical depth; (ii) map these depth surfaces onto cytoarchitectonic laminae using BigBrain-derived laminar boundaries; (iii) systematically quantify the SNR, coregistration, cortical-thickness, lead-field, and orientation constraints governing multilayer inference; (iv) evaluate failure modes including patch-size mismatch and simultaneous sources at different depths; and (v)

apply the framework to three independent empirical ERF datasets to test whether expected laminar activation motifs can be recovered in visual, motor, and somatosensory cortex. Prior work was limited to binary deep-versus-superficial comparisons and did not attempt depth-resolved inference across intermediate cortical depths or cytoarchitectonic laminae. Importantly, because all cortical depth surfaces are now jointly downsampled while preserving one-to-one vertex correspondence, each layer-specific source model contained the same number of dipoles and free parameters. Furthermore, dipole orientations were constrained using link vectors between corresponding vertices across surfaces. Consequently, differences in free energy between layer models reflect differences in source depth and associated lead fields, rather than differences in model complexity, source-space dimensionality, or orientation priors.

We also agree that the empirical data cannot, in the absence of invasive ground truth, validate accurate reconstruction of individual laminae in the same way as simulations. We have therefore revised the manuscript to distinguish more clearly between what is demonstrated by simulations and what is supported empirically. The simulations establish the conditions under which multilayer laminar inference is theoretically feasible when depth is the principal factor distinguishing competing source models, whereas the empirical analyses demonstrate recovery of expected coarse laminar-family dynamics in well-characterized ERFs. Accordingly, we have softened the title, abstract, and relevant claims throughout the manuscript, replacing language such as "inference across all six cortical laminae" and "laminar precision is achievable" with more conservative phrasing emphasizing "depth-resolved laminar inference under favorable conditions" and "extension beyond deep-versus-superficial distinctions."

Abstract:

Neural dynamics at the laminar level are critical for cortical computation. However, in humans, non-invasive methods to probe such dynamics have been limited to coarse distinctions between deep and superficial layers. Here, we present a multilayer magnetoencephalography source reconstruction framework and evaluate the conditions under which depth-resolved laminar inference may be feasible. Using simulations, we systematically assess the limits of magnetoencephalography depth resolution, showing that laminar discrimination depends on sufficiently high signal-to-noise ratio, precise co-registration, and accurate specification of cortical column orientation. We demonstrate that regional variations in cortical anatomy influence reconstruction fidelity, with lead-field separability emerging as a key determinant. We then apply this framework to empirical data from three independent datasets and find laminar activation patterns that align with canonical feedforward and feedback motifs in visual and sensorimotor circuits, supporting the plausibility of laminar inference under favorable conditions and offering opportunities to bridge invasive electrophysiology and human neuroimaging.

We have added text to the Introduction and Discussion clarifying this distinction and explicitly stating the novelty relative to previous work.
Introduction (lines 85-110):

Here, we extend these approaches by introducing a multilayer hpMEG source reconstruction framework that moves beyond binary deep-versus-superficial comparisons toward depth-resolved inference across the cortical column. We expand the source space to include an arbitrary number of intermediate layers. We refer to the equidistant depth surfaces used in our forward models as layers (denoted with Arabic numerals, i.e. 1-11), while we use laminae (denoted with Roman numerals) to refer to cytoarchitectonically defined cortical layers (i.e. I-VI). We first use simulations to systematically characterize the conditions under which laminar inference is feasible, quantifying the influence of SNR, co-registration accuracy, dipole orientation, and anatomical variability across the cortex. This extends prior work by moving from two-layer model comparison[20] to a finer-grained depth-resolved framework and by explicitly identifying the anatomical and data-quality constraints that govern inference accuracy.

Importantly, we distinguish between theoretical feasibility and empirical validation. While simulations allow assessment of laminar inference accuracy under controlled conditions, empirical data lack ground-truth laminar labels. Accordingly, our empirical analyses focus on whether multilayer hpMEG recovers expected laminar-family activation patterns in well-characterized event-related fields (ERFs). In the visual cortex, hpMEG recovers a feedforward activation sequence consistent with laminar models of sensory processing. In the somatosensory and motor cortices, analysis of two independent datasets reveals broadly consistent deep-superficial activation patterns characteristic of motor execution. These results demonstrate that multilayer hpMEG extends non-invasive laminar inference beyond coarse depth distinctions under favorable conditions, while highlighting the constraints that currently limit precise identification of individual laminae *in vivo*. This positions hpMEG as a powerful tool for interrogating neural circuits at a laminar level, offering new opportunities to study how computations within and across cortical laminae shape network-level interactions underlying cognition and behavior[6].

Discussion (lines 480-521):

Building upon previous models of laminar inference in high-precision MEG (hpMEG) that compared deep versus superficial sources, we introduce a multilayer source reconstruction framework that aims to enable depth-resolved inference across the cortical column. Simulations establish feasibility limits for laminar inference, anatomical constraints guide vertex selection, and Bayesian model comparison provides statistical inference over cortical depth. In the simulations, this precision required a sufficiently high signal-to-noise ratio (approximately -35 dB for deep-versus-superficial distinctions and -20 dB for full laminar inference) and minimal co-registration error (< 2mm), conditions that can be met in practice through the use of individualized head-casts and optimized recording protocols[21,22]. While coarse deep-versus-superficial distinctions remain feasible at lower SNRs with two-layer source models, resolving finer laminar structure requires sufficiently high SNR, precise co-registration, and accurate forward modeling, and

should therefore be interpreted as conditional on these factors. Importantly, supplementary robustness simulations demonstrated that these conclusions were largely preserved under more realistic conditions, including temporally autocorrelated noise, concurrent interfering sources, source-space mismatch, and forward-model mismatch, although the latter reduced the range of SNRs and co-registration errors over which accurate laminar inference could be achieved. Crucially, we confirm that correctly estimating cortical column orientation is essential for accurate laminar inference, as errors in dipole orientation estimation systematically degrade localization accuracy. Corroborating previously established results[20], we show that anatomical features of the cortex such as thickness, dipole orientation, scalp-to-cortex distance, and lead field separation across depths nonlinearly shape the spatial distribution of inference accuracy, with lead field separability emerging as the strongest predictor.

Beyond simulations, we demonstrate the empirical plausibility of multilayer hpMEG by examining whether it recovers laminar activation patterns in human visual and sensorimotor cortex that are consistent with established physiological models. Notably, visual and motor ERFs exhibited complementary laminar sequences, with early granular and supragranular dominance in visual cortex and early infragranular dominance in motor cortex, reflecting their distinct circuit architectures and afferent/efferent nature, respectively. In primary visual cortex, visually evoked fields exhibited a feedforward laminar progression, with early granular dominance consistent with thalamocortical input, followed by supragranular activity at the response peak, with the same feedforward laminar sequence observed in both contralateral and ipsilateral hemispheres. In motor circuits, combining two independent datasets revealed consistent laminar trends across datasets: motor fields in primary motor cortex were dominated by infragranular activity beginning before movement onset and recurring around execution, consistent with corticospinal output, followed by delayed supragranular responses, while motor-evoked responses in primary somatosensory cortex showed predominant superficial activity at movement onset, consistent with somatosensory reafference. The convergence of these laminar patterns across cortical systems, task variants, and acquisition sites suggests that multilayer hpMEG can recover established physiological motifs at the level of laminar families under favorable conditions, providing evidence that non-invasive laminar inference can extend beyond deep-versus-superficial distinctions.

lines 735-750:

In summary, we provide a framework for multilayer hpMEG and define the conditions under which depth-resolved laminar inference may be feasible. By systematically exploring the trade-offs between signal quality, anatomical constraints, and inference accuracy, we delineate best-case and marginal conditions for MEG-based laminar inference. While current implementations reliably support deep-versus-superficial distinctions and laminar-family-level interpretations, resolving individual laminae *in vivo* remains contingent on favorable conditions and requires further validation. These findings further suggest that there is unlikely to be a universal minimum trial count required for laminar inference. Instead, the achievable precision will depend on the

interaction between trial count, component magnitude and variability, preprocessing quality, head stabilization, and recording noise characteristics, all of which determine the effective SNR of the evoked response. Future developments, including Bayesian model averaging, individualized laminar and orientation priors, more accurate forward models, and emerging sensor technologies, may further improve hpMEG's ability to resolve cortical computations at the laminar level. These advances pave the way for a new era of laminar human neuroscience, bridging invasive electrophysiology with whole-brain, millisecond-resolution functional imaging.

2. The strongest evidence regarding the ability to resolve individual cortical laminae comes from the simulations. However, simulation results can not be directly extrapolated to real data for the following reasons:

   a. Real noise is more complex than the simulated white noise.

   b. Their simulations do not account for errors in the forward model. As far as I can tell, this point is not even discussed. They use a relatively simple single-shell forward model. Since it is used in the simulation for forward and inverse modeling it does not introduce errors. However, in real data, multilayer results will most likely strongly depend on the accuracy of the forward model. More realistic forward models (like multi-compartment FEM models) might be needed. This is a critical point since their inference is based on leadfield differences between layers and the leadfield computation is based on the volume conductor model (single-shell, BEM, FEM).

   c. Simulations are based on single sources (with one exception) and all conclusions regarding minimal SNR and coregistration accuracy are entirely based on single sources. This is a scenario that does not exist in the brain! Therefore, in real data, multilayer results might be affected by nearby sources.

   All these points should at least be discussed.

We agree that the original simulations represented an idealized scenario and that the ability to resolve individual cortical laminae in empirical data depends on factors beyond those captured by the initial simulations. To address these concerns, we performed four additional sets of robustness analyses. First, to evaluate the impact of more realistic noise structure, we repeated the SNR and co-registration simulations using temporally autocorrelated pink (1/f) noise rather than white noise. These simulations produced qualitatively similar results, with only minor changes in reconstruction accuracy and bias. Second, to assess the effect of concurrent neural activity, we repeated the simulations with three additional interfering sources located at different cortical positions. Although reconstruction accuracy was modestly reduced relative to the single-source simulations, the principal conclusions regarding SNR and co-registration requirements remained unchanged. Third, to assess robustness to forward-model mismatch, we performed simulations using an alternative generative model (AGM) in which source activity was simulated on a cortical source space generated using a different surface discretization than that used for inversion. Although reconstruction accuracy was modestly reduced, the overall dependence on SNR and co-registration precision remained qualitatively similar to the original simulations. Fourth,

because forward-model accuracy is central to laminar inference, we performed additional simulations in which data were generated using a boundary element model (BEM) forward model but reconstructed using the Nolte single-shell model employed throughout the manuscript. These simulations again showed the same overall trends, although the range of SNRs and co-registration errors over which accurate laminar inference was possible was reduced. This finding is consistent with the reviewer's observation that depth-resolved source reconstruction depends critically on the accuracy of the forward model. More realistic forward models, such as BEM or FEM approaches, may therefore further improve achievable laminar precision in empirical applications. However, the present results demonstrate that the principal conclusions remain qualitatively robust even under substantial forward-model mismatch.

With regards to concurrent neural sources, several aspects of the empirical analysis further mitigate this potential issue. First, the analyses were performed on trial-averaged ERFs, which preferentially retain activity that is temporally locked and phase-consistent across trials while suppressing unrelated ongoing activity. Second, laminar inference was performed within short sliding windows (10-50 ms), reducing temporal overlap between distinct response components. Third, MSP inversion was constrained to a localized cortical patch centered on a functionally selected vertex, limiting ambiguity from spatially distributed activity. Nonetheless, the equal-strength two-source simulations demonstrate that simultaneous generators at different cortical depths can bias inference toward intermediate layers, indicating that the current framework is best interpreted as identifying the dominant laminar contribution within a localized cortical region and time window, rather than fully resolving multiple concurrent laminar generators independently.

We have added a new Results subsection summarizing these robustness analyses and corresponding supplementary figures 6-13). Across all conditions, the largest reductions in performance were observed when the generative model differed from the inverse model, highlighting that forward-model accuracy is likely to be a primary determinant of achievable laminar precision. This observation also suggests that future implementations using more realistic BEM or FEM forward models may further improve performance. Nonetheless, the principal conclusions of the study remained qualitatively unchanged across all robustness analyses. We also expanded the Discussion to explicitly address the dependence of laminar inference on forward-model accuracy and the potential influence of multiple concurrent sources.

We describe these simulations in the Methods section (lines 877-898):

To assess the extent to which the primary simulation results depended on simplifying assumptions, we performed four additional robustness analyses. First, the SNR and co-registration simulations were repeated using temporally autocorrelated pink noise (1/f amplitude spectrum) generated independently at each sensor and scaled to the same target RMS noise level as the original white-noise simulations. Second, to assess robustness to concurrent activity, three additional interfering sources were added at randomly selected cortical locations in the middle cortical layer. These sources had

independent band-limited temporal profiles (10-30 Hz) and amplitudes equal to 10% of the primary simulated source. Third, to evaluate the effect of source-space mismatch, simulations were generated using an alternative generative model (AGM)[34] based on multilayer cortical surfaces containing 32,399 vertices per layer, whereas source reconstruction was performed using the source model surfaces employed throughout the main analyses (29,130 vertices per layer), generated using a different surface downsampling factor (11% rather than 10%). This resulted in systematic differences between simulated dipole locations and the source locations assumed during inversion. Finally, to assess the effect of forward-model mismatch, simulated data were generated using lead fields computed with a boundary element model (BEM) forward model (computed from subject-specific inner-skull, outer-skull, and scalp surfaces using OpenMEEG v2.1.0[35]), while source reconstruction was performed using the Nolte single-shell model used throughout the study. For each robustness analysis, the same SNR and co-registration error manipulations as in the primary simulations were applied, and reconstruction accuracy, bias, and model-evidence structure were evaluated using identical procedures (see Simulation Analysis, below).

and the results of these simulations in the new Results subsection (lines 314-347):

Robustness to noise, model mismatch, and concurrent sources

To assess the extent to which the simulation results generalize beyond the idealized conditions of the primary analyses, we performed a series of robustness analyses that relaxed key assumptions regarding noise structure, forward-model accuracy, and source complexity (Supplementary Fig. 6-13). First, we repeated the SNR and co-registration simulations using temporally autocorrelated pink noise rather than white noise. Reconstruction accuracy, bias, and free-energy structure were largely unchanged, indicating that the principal findings do not depend on the assumption of spectrally flat sensor noise Supplementary Fig. 6 and 10). Second, we evaluated robustness to concurrent activity by introducing three additional interfering sources at random cortical locations. Each interfering source had an independent band-limited temporal profile and an amplitude equal to 10% of the primary source. Although reconstruction accuracy was modestly reduced, reliable laminar inference still emerged between approximately -35 and -20 dB SNR and deteriorated beyond approximately 2-3 mm co-registration error, closely matching the results of the primary simulations (Supplementary Fig. 7 and 11). Third, we assessed robustness to source-space mismatch using an alternative generative model in which simulated activity was generated on cortical surfaces containing 32,399 vertices per layer, whereas inversion was performed using the source model employed throughout the manuscript (29,130 vertices per layer). This mismatch resulted in modest reductions in reconstruction accuracy but preserved the overall dependence on SNR and co-registration precision (Supplementary Fig. 8 and 12). Finally, because laminar inference relies on subtle differences in lead fields across cortical depth, we evaluated the effect of forward-model mismatch by generating data using a BEM forward model while reconstructing sources using the Nolte single-shell model used throughout the main analyses. This manipulation produced the largest

degradation in performance among the robustness analyses, reducing the range of SNRs and co-registration errors over which accurate laminar inference could be achieved. Nevertheless, the overall structure of the results remained similar to the primary simulations, with reconstruction accuracy improving monotonically with increasing SNR and decreasing co-registration error (Supplementary Fig. 9 and 13). To summarize these analyses, Figure 7 compares mean reconstruction error across all robustness conditions. Although performance was reduced under model mismatch and BEM-generated data, the qualitative conclusions regarding the requirements for reliable laminar inference remained unchanged. Collectively, these results indicate that the principal findings are robust to realistic deviations from the assumptions of the primary simulations, while highlighting the importance of accurate forward modeling for depth-resolved source reconstruction.

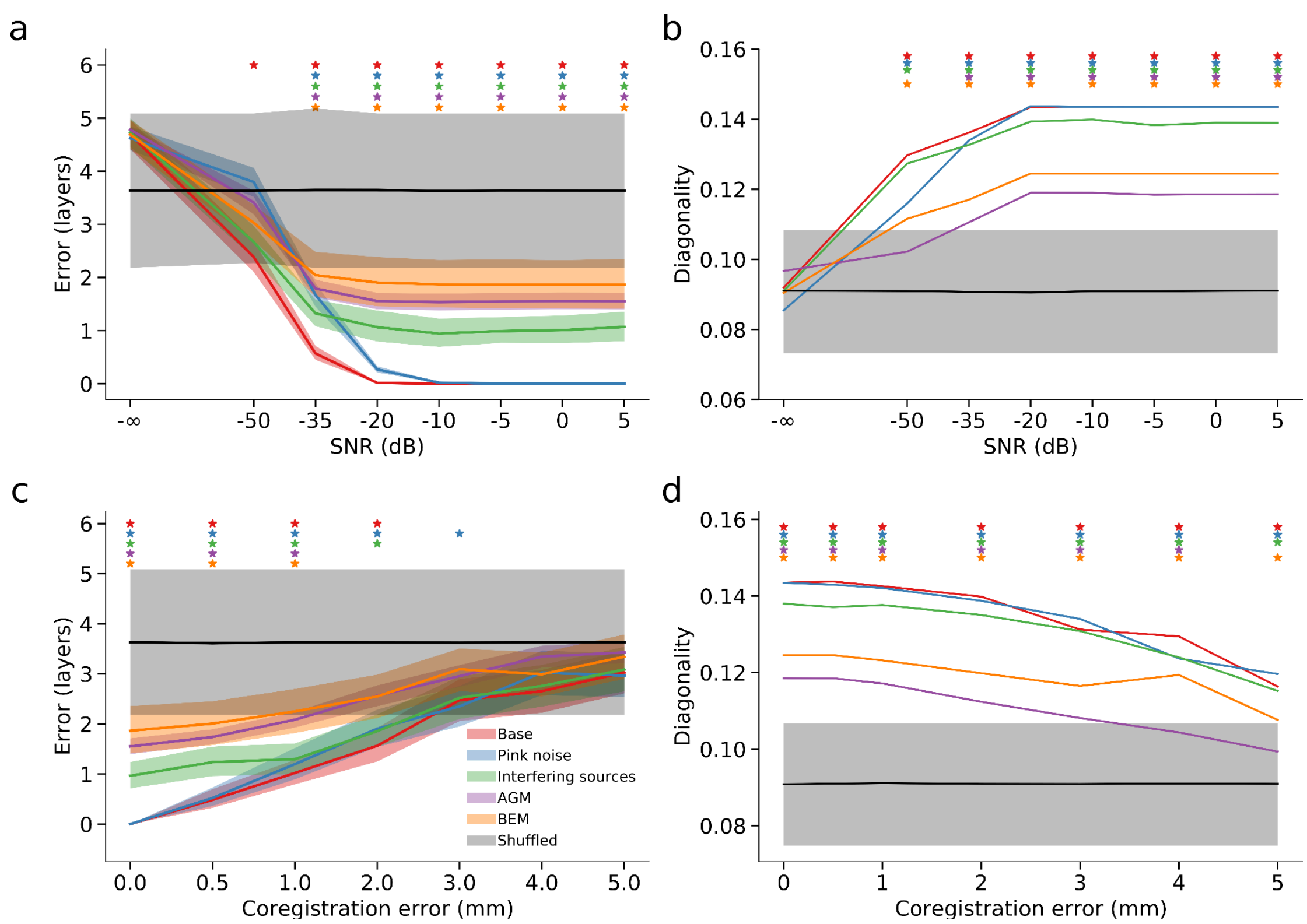

Figure 7. **Robustness of laminar source reconstruction to violations of simulation assumptions.** Comparison of reconstruction performance across the primary simulations (base) and four robustness analyses: pink noise, interfering sources, alternative generative model (AGM), and boundary element model (BEM) generative model. The AGM simulations used mismatched cortical source-space discretizations for simulation and inversion, whereas the BEM simulations used a BEM forward model for data generation and a Nolte single-shell model for source reconstruction. **a** Mean laminar reconstruction error (layers) for simulated versus shuffled data a function of SNR. **b** Diagonal dominance of the mean model-evidence matrices (trace/sum) for simulated versus shuffled data as a function of SNR. **c** Mean laminar reconstruction error (layers) for simulated versus shuffled data as a function of co-registration error. **d** Diagonal dominance of the mean model-evidence matrices (trace/sum) for simulated versus shuffled data as a function of co-registration error. Shaded regions indicate ± SEM across simulated cortical locations. Colored asterisks denote simulation conditions in which performance differed significantly from chance ($p < 0.05$). Across all robustness analyses, the qualitative dependence of laminar inference on SNR and co-registration precision was preserved. Pink-noise simulations produced results nearly identical to the primary simulations, whereas interfering sources caused only modest reductions in performance. The largest decreases in reconstruction accuracy and matrix diagonal dominance were observed for simulations incorporating forward-model mismatch (AGM and BEM).

Supplementary Material:

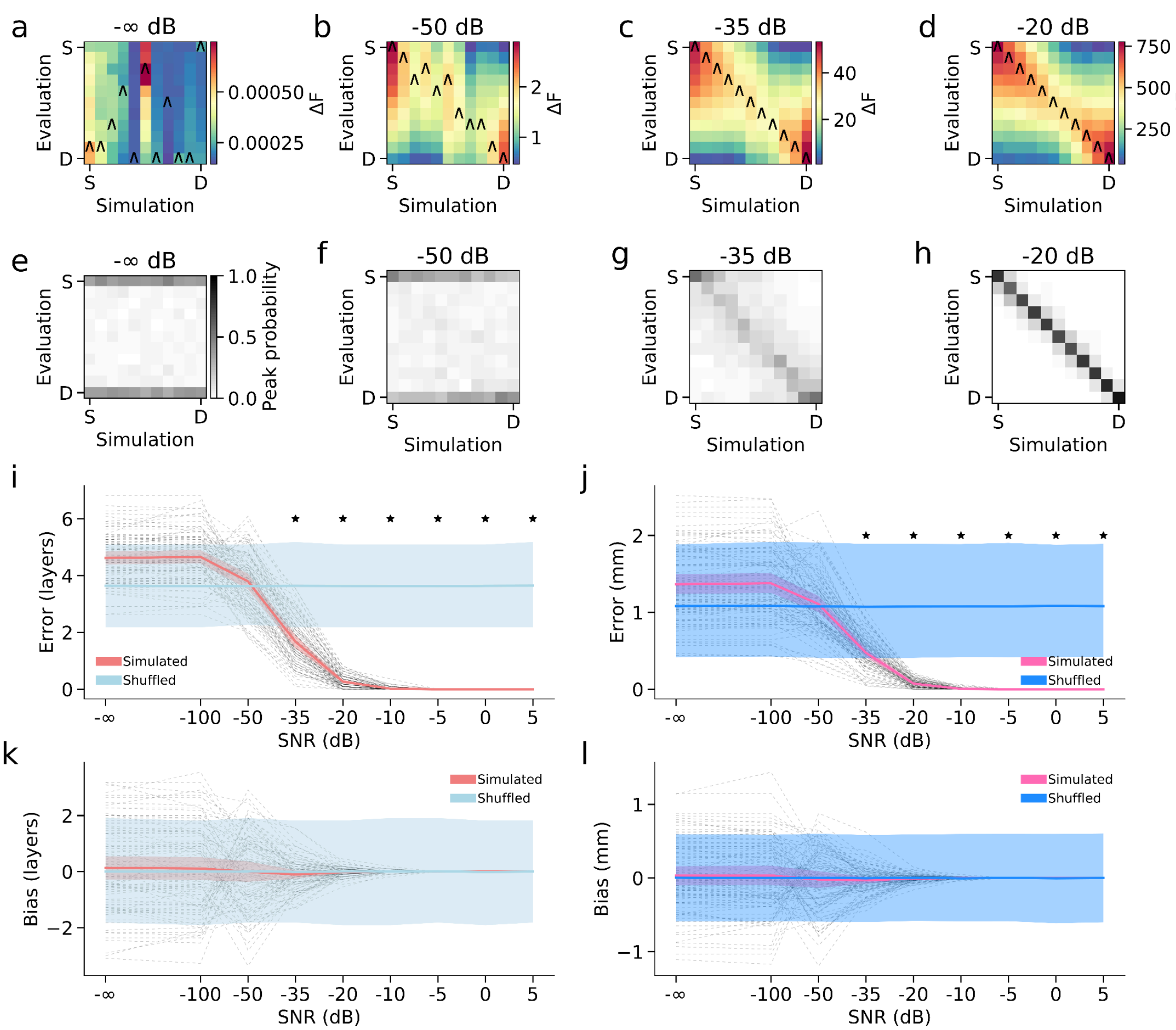

Supplementary Figure 6. **Laminar source-reconstruction accuracy as a function of SNR using pink noise. a-d** Mean model evidence (free energy, ΔF) matrices (relative to the worst

model; S = superficial surface layer, D = deep surface layer) for simulated signals placed on 11 cortical surfaces at four SNR levels (-∞ dB, -50 dB, -35 dB, -20 dB). Caret markers denote the forward model with peak free energy for each simulated depth. **e-h** Probability density of each forward model being the maximum-evidence solution, aggregated across cortical locations. **i, j** Laminar reconstruction error (in layers and mm). **k, l** Laminar reconstruction bias (in surface layers and millimeters), again comparing simulated and shuffled data. Shaded bands represent 95% confidence intervals, and asterisks mark SNR levels at which observed error or bias differed significantly from chance ($p < 0.05$).

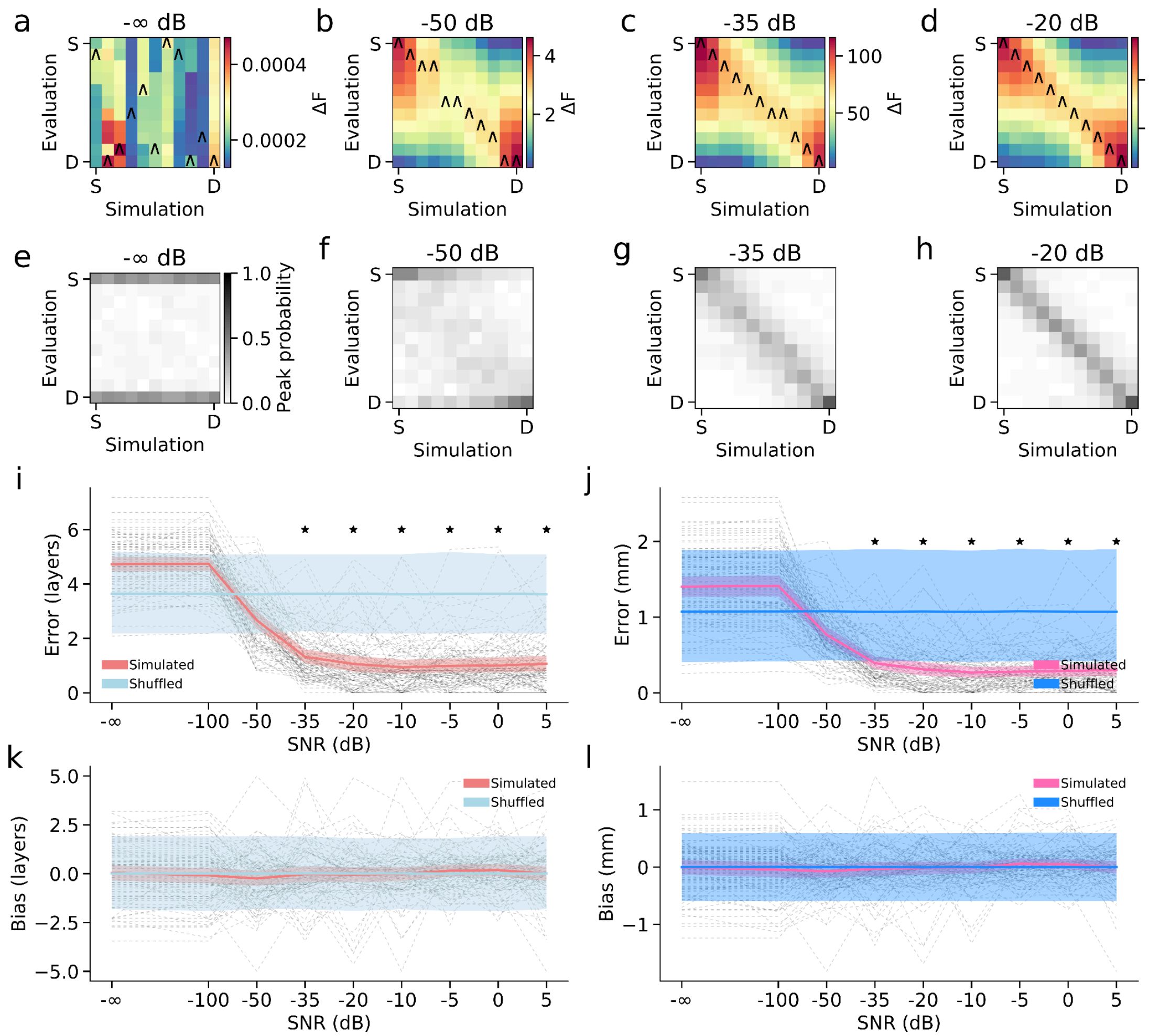

Supplementary Figure 7. **Laminar source-reconstruction accuracy as a function of SNR in the presence of additional interfering sources.** Simulations included three simultaneous distractor sources placed at random cortical locations in the middle cortical surface, each with 10% of the amplitude of the primary simulated source and band-limited (10-30 Hz) temporal profiles. **a-d** Mean model evidence (free energy, ΔF) matrices (relative to the worst model; S = superficial surface layer, D = deep surface layer) for simulated signals placed on 11 cortical surfaces at four SNR levels (-∞ dB, -50 dB, -35 dB, -20 dB). Caret markers denote the forward model with peak free energy for each simulated depth. **e-h** Probability density of each forward model being the maximum-evidence solution, aggregated across cortical locations. **i, j** Laminar reconstruction error (in layers and mm). **k, l** Laminar reconstruction bias (in surface layers and millimeters), again comparing simulated and shuffled data. Shaded bands represent 95% confidence intervals, and asterisks mark SNR levels at which observed error or bias differed significantly from chance ($p < 0.05$).

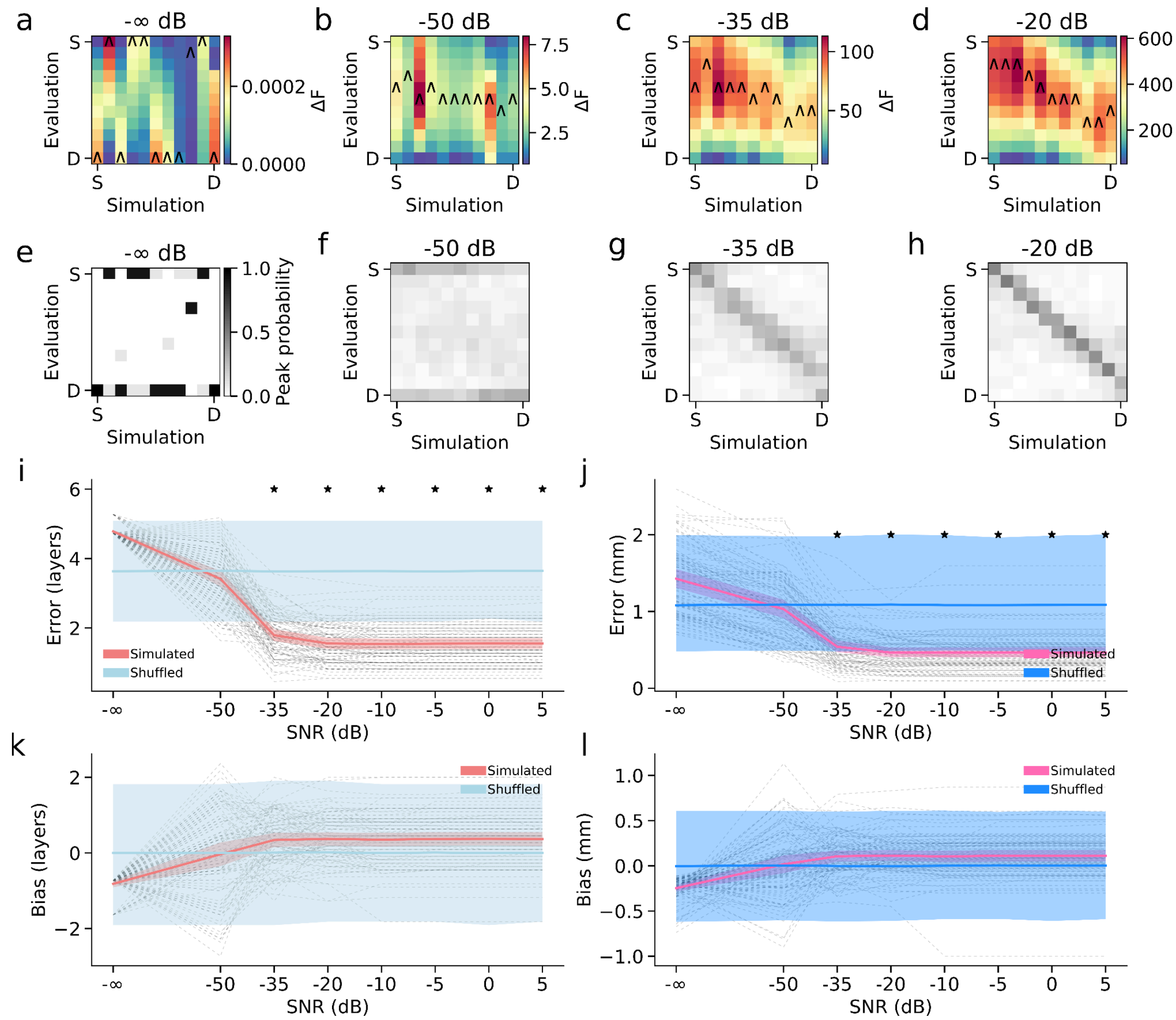

Supplementary Figure 8. **Laminar source-reconstruction accuracy as a function of SNR using mismatched cortical source spaces for simulation and inversion.** Simulated data were generated using multilayer cortical surfaces containing 32,399 vertices per layer, whereas source reconstruction was performed using the cortical source model employed in the main analyses (29,130 vertices per layer), introducing systematic mismatch between simulated and reconstructed dipole locations. **a-d** Mean model evidence (free energy, ΔF) matrices (relative to the worst model; S = superficial surface layer, D = deep surface layer) for simulated signals placed on 11 cortical surfaces at four SNR levels (-∞ dB, -50 dB, -35 dB, -20 dB). Caret markers denote the forward model with peak free energy for each simulated depth. **e-h** Probability density of each forward model being the maximum-evidence solution, aggregated across cortical locations. **i, j** Laminar reconstruction error (in layers and mm). **k, l** Laminar reconstruction bias (in surface layers and millimeters), again comparing simulated and shuffled data. Shaded bands represent 95% confidence intervals, and asterisks mark SNR levels at which observed error or bias differed significantly from chance ($p < 0.05$).

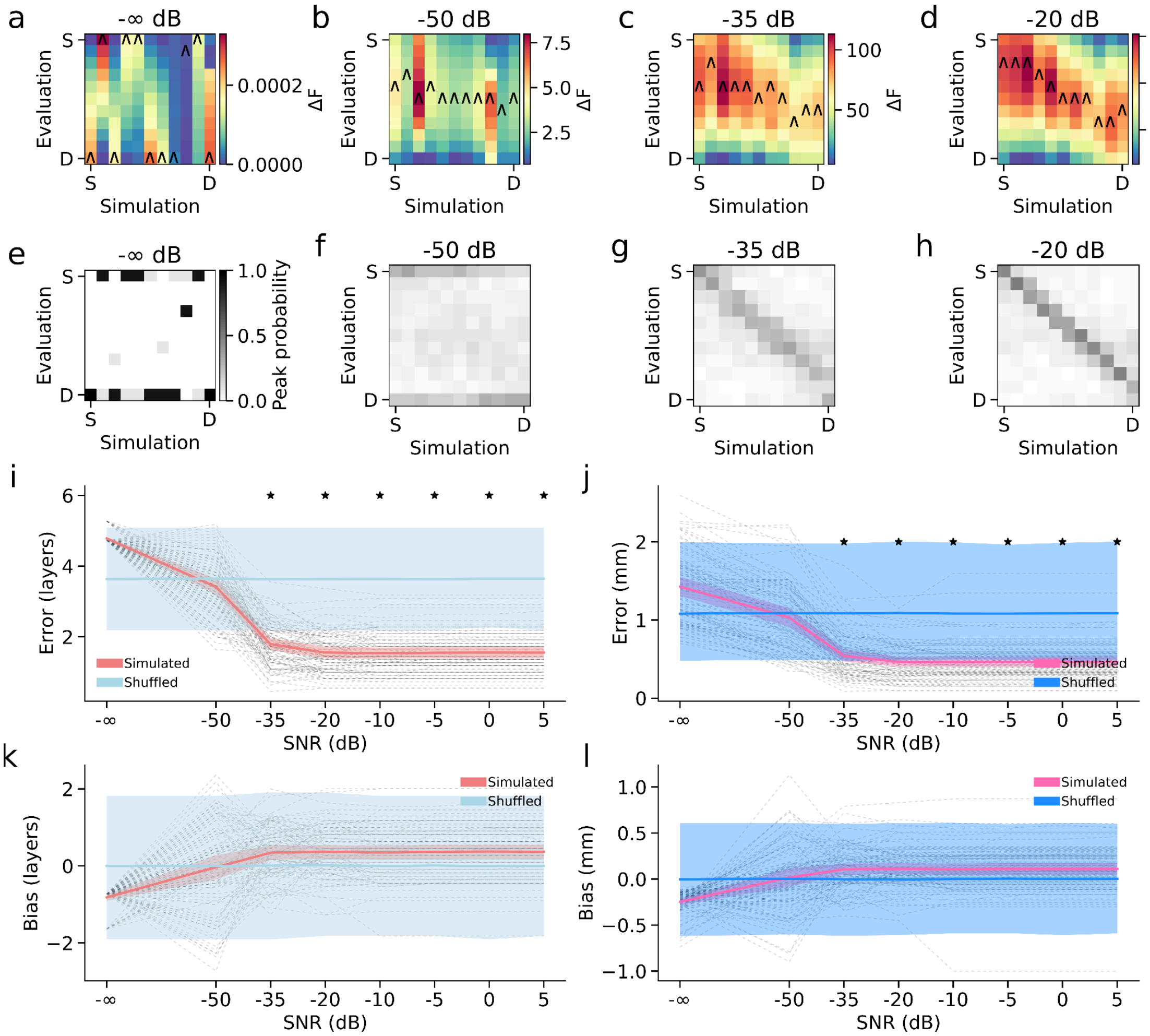

Supplementary Figure 9. **Laminar source-reconstruction accuracy as a function of SNR using mismatched forward models.** Simulated data were generated using lead fields from a boundary element model (BEM) forward model, whereas source reconstruction was performed using the same Nolte single shell forward model employed in the main analyses. **a-d** Mean model evidence (free energy, ΔF) matrices (relative to the worst model; S = superficial surface layer, D = deep surface layer) for simulated signals placed on 11 cortical surfaces at four SNR levels (-∞ dB, -50 dB, -35 dB, -20 dB). Caret markers denote the forward model with peak free energy for each simulated depth. **e-h** Probability density of each forward model being the maximum-evidence solution, aggregated across cortical locations. **i, j** Laminar reconstruction error (in layers and mm). **k, l** Laminar reconstruction bias (in surface layers and millimeters), again comparing simulated and shuffled data. Shaded bands represent 95% confidence intervals, and asterisks mark SNR levels at which observed error or bias differed significantly from chance ($p < 0.05$).

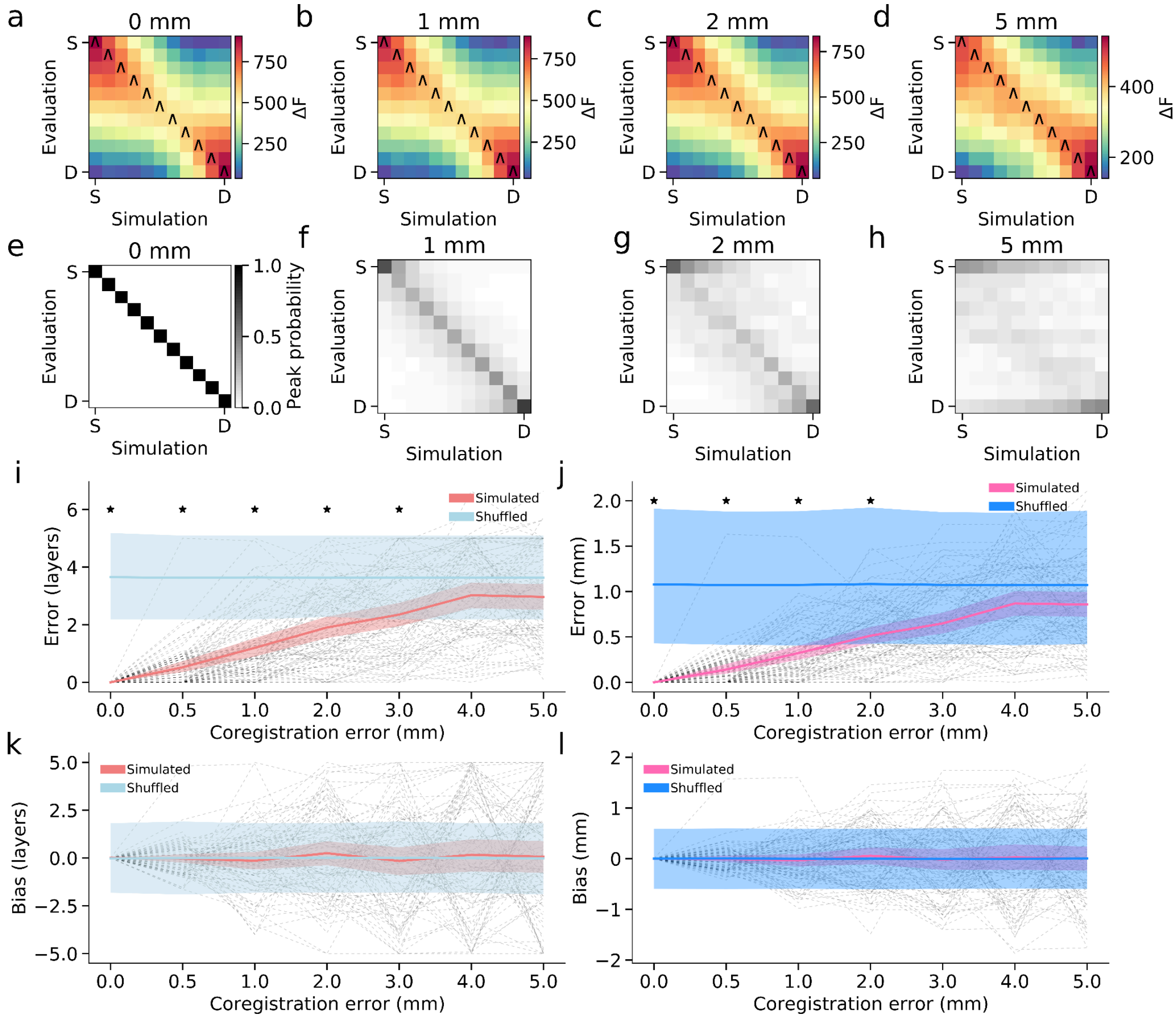

Supplementary Figure 10. **Laminar source-reconstruction accuracy as a function of co-registration error using pink noise. a-d** Mean model evidence (free energy, ΔF) matrices (relative to the worst model; S = superficial surface layer, D = deep surface layer) for simulated signals placed on 11 cortical surfaces 0, 1, 2, and 5 mm co-registration error. Caret markers denote the forward model with peak free energy for each simulated depth. **e-h** Probability density of each forward model being the maximum-evidence solution, aggregated across cortical locations. **i, j** Laminar reconstruction error (in layers and mm). **k, l** Laminar reconstruction bias (in surface layers and millimeters), again comparing simulated and shuffled data. Shaded bands represent 95% confidence intervals, and asterisks mark significance at $p < 0.05$.

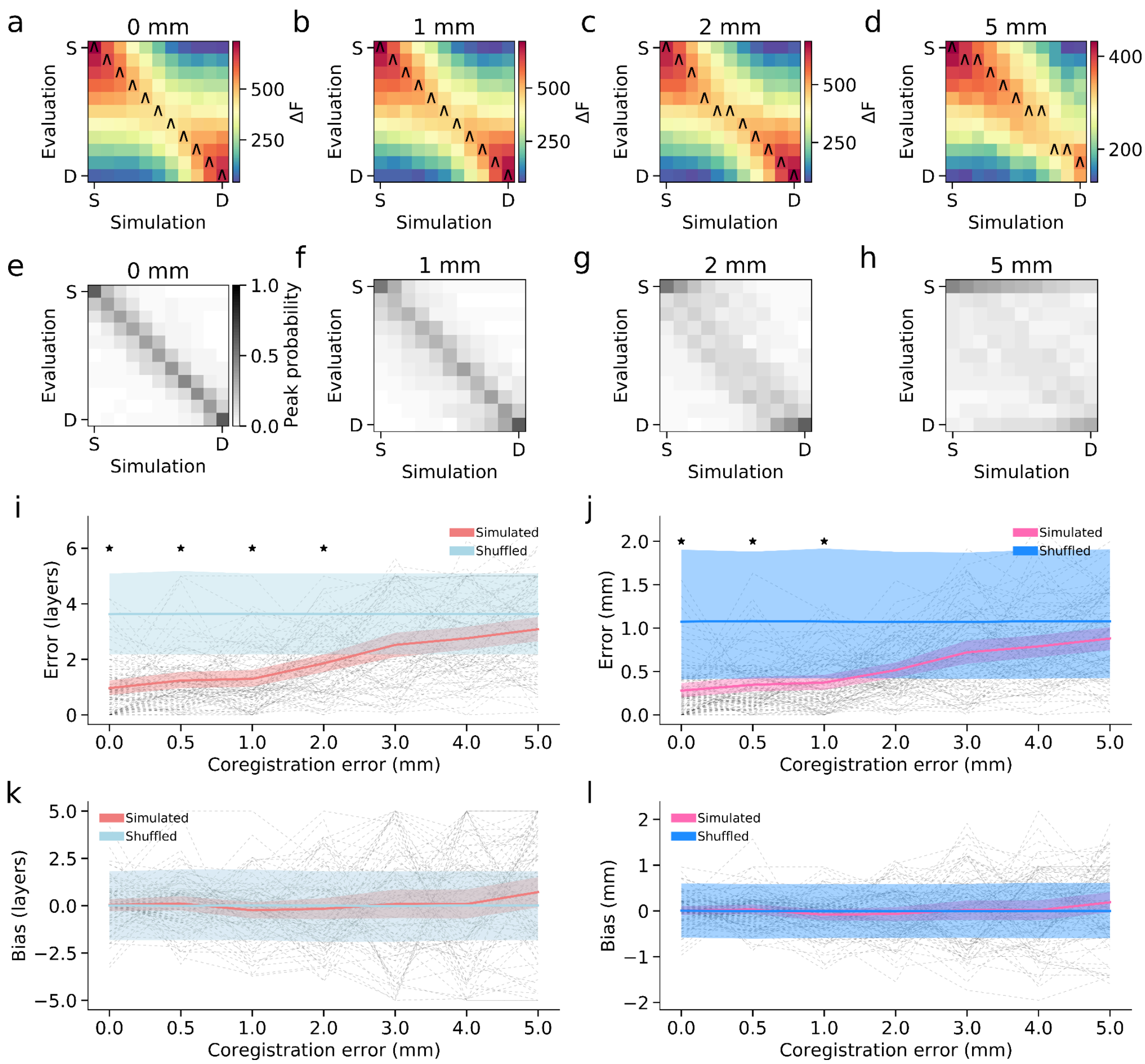

Supplementary Figure 11. **Laminar source-reconstruction accuracy as a function of co-registration error in the presence of additional interfering sources.** Simulations included three simultaneous distractor sources placed at random cortical locations in the middle cortical surface, each with 10% of the amplitude of the primary simulated source and band-limited (10-30 Hz) temporal profiles. **a-d** Mean model evidence (free energy, ΔF) matrices (relative to the worst model; S = superficial surface layer, D = deep surface layer) for simulated signals placed on 11 cortical surfaces 0, 1, 2, and 5 mm co-registration error. Caret markers denote the forward model with peak free energy for each simulated depth. **e-h** Probability density of each forward model being the maximum-evidence solution, aggregated across cortical locations. **i, j** Laminar reconstruction error (in layers and mm). **k, l** Laminar reconstruction bias (in surface layers and millimeters), again comparing simulated and shuffled data. Shaded bands represent 95% confidence intervals, and asterisks mark significance at $p < 0.05$.

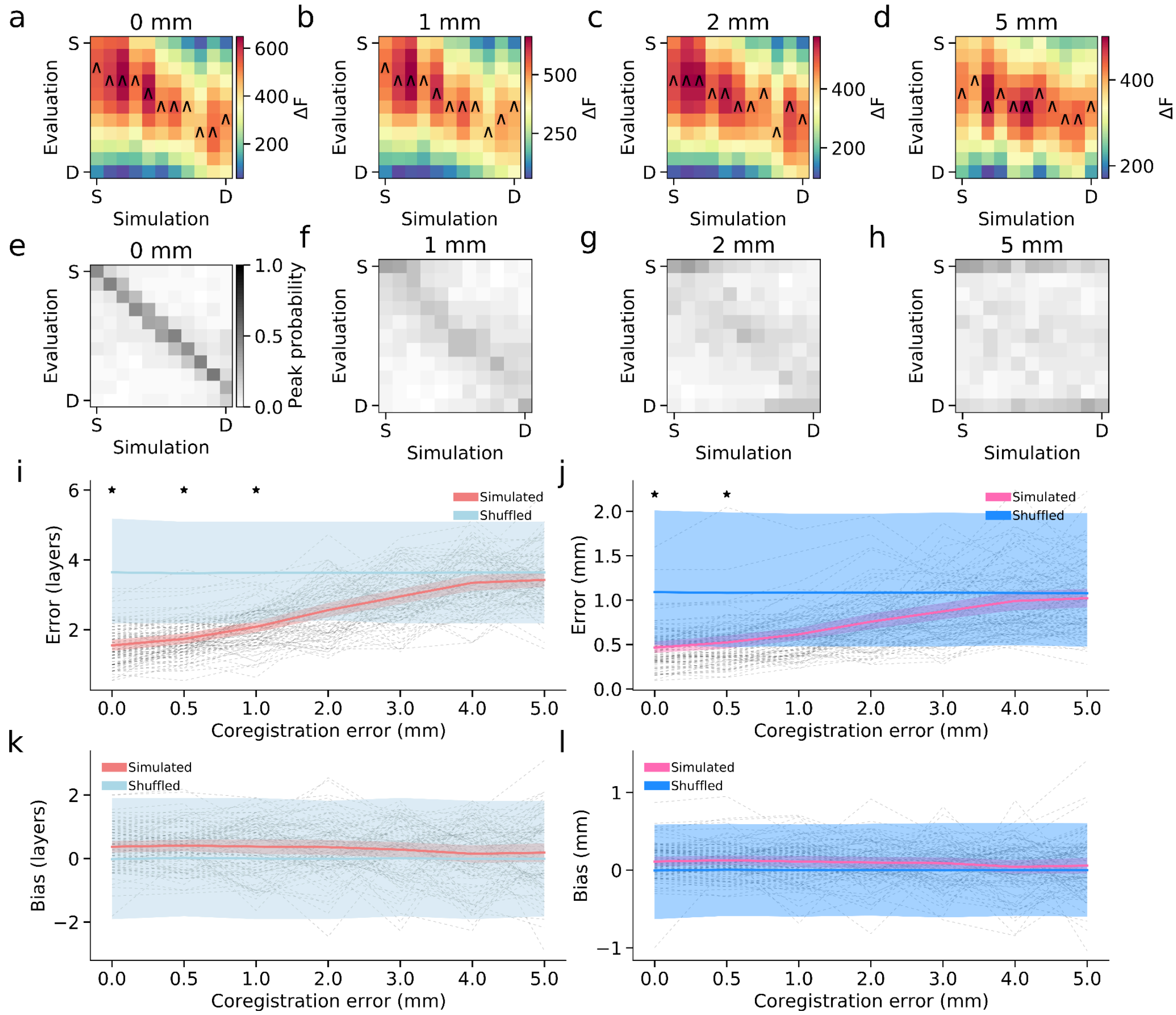

Supplementary Figure 12. **Laminar source-reconstruction accuracy as a function of co-registration error using mismatched cortical source spaces for simulation and inversion.** Simulated data were generated using multilayer cortical surfaces containing 32,399 vertices per layer, whereas source reconstruction was performed using the cortical source model employed in the main analyses (29,130 vertices per layer), introducing systematic mismatch between simulated and reconstructed dipole locations. **a-d** Mean model evidence (free energy, ΔF) matrices (relative to the worst model; S = superficial surface layer, D = deep surface layer) for simulated signals placed on 11 cortical surfaces 0, 1, 2, and 5 mm co-registration error. Caret markers denote the forward model with peak free energy for each simulated depth. **e-h** Probability density of each forward model being the maximum-evidence solution, aggregated across cortical locations. **i, j** Laminar reconstruction error (in layers and mm). **k, l** Laminar reconstruction bias (in surface layers and millimeters), again comparing simulated and shuffled data. Shaded bands represent 95% confidence intervals, and asterisks mark significance at $p < 0.05$.

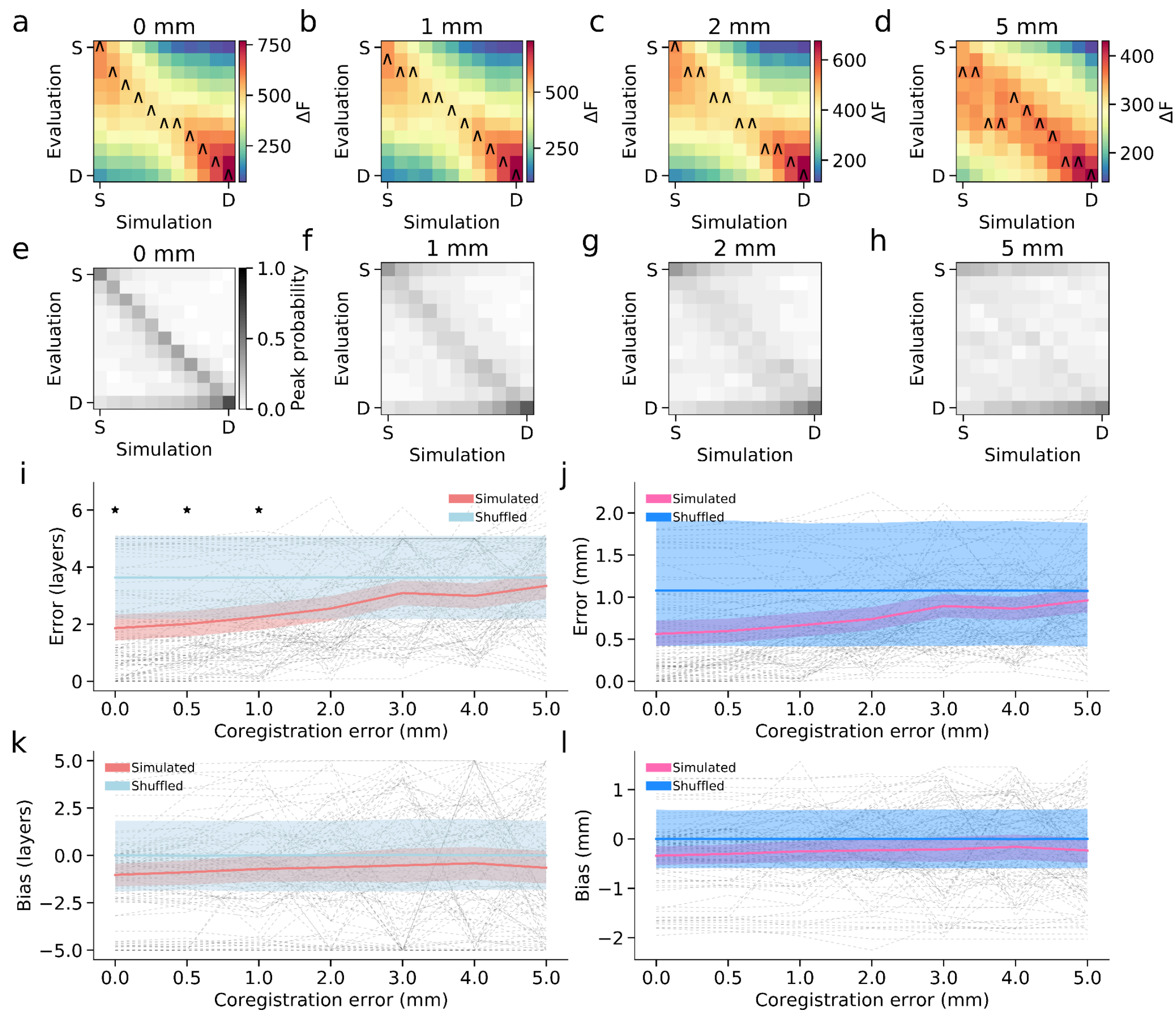

Supplementary Figure 13. **Laminar source-reconstruction accuracy as a function of co-registration error using mismatched forward models.** Simulated data were generated using lead fields from a boundary element model (BEM) forward model, whereas source reconstruction was performed using the same Nolte single shell forward model employed in the main analyses. **a-d** Mean model evidence (free energy, ΔF) matrices (relative to the worst model; S = superficial surface layer, D = deep surface layer) for simulated signals placed on 11 cortical surfaces 0, 1, 2, and 5 mm co-registration error. Caret markers denote the forward model with peak free energy for each simulated depth. **e-h** Probability density of each forward model being the maximum-evidence solution, aggregated across cortical locations. **i, j** Laminar reconstruction error (in layers and mm). **k, l** Laminar reconstruction bias (in surface layers and millimeters), again comparing simulated and shuffled data. Shaded bands represent 95% confidence intervals, and asterisks mark significance at $p < 0.05$.

We now have updated the Discussion section of the the manuscript accordingly (lines 492-496):

Importantly, supplementary robustness simulations demonstrated that these conclusions were largely preserved under more realistic conditions, including temporally autocorrelated noise, concurrent interfering sources, source-space mismatch, and forward-model mismatch, although the latter reduced the range of SNRs and co-registration errors over which accurate laminar inference could be achieved.

lines 522-594:

The empirical results demonstrate that multilayer hpMEG can recover physiologically plausible laminar activation sequences in both visual and sensorimotor cortices. At the same time, the simulations highlight important constraints on the interpretation of these findings. Laminar inference depends critically on the accuracy of the underlying forward model, is best interpreted as identifying the dominant laminar contribution within a localized cortical region and time window rather than fully resolving multiple simultaneous generators, and remains sensitive to methodological choices such as patch size, sliding-window duration, and preprocessing.

Accurate laminar inference ultimately depends on the fidelity of the forward model. A potential concern is that monotonic variations in peak model evidence across cortical depth could reflect depth-dependent biases in the forward model, signal-to-noise gradients, or regularization effects rather than true laminar specificity. However, our interpretation does not rely on static depth profiles at a single latency, but on the temporal evolution of laminar model evidence, which shows systematic shifts in dominant laminar contributions over time. Such dynamic transitions are not readily explained by fixed biases. Moreover, because the layer-specific source models are matched in complexity and orientation priors through vertex correspondence and link vector constraints, differences in model evidence primarily reflect depth-dependent differences in the forward model rather than differences in model dimensionality. Nonetheless, resolving activity from intermediate laminae remains challenging, particularly under lower SNR conditions. The simulations further demonstrated that lead field separability between superficial and deep cortical layers is the strongest anatomical predictor of laminar inference accuracy, emphasizing the importance of accurate forward modeling. To assess robustness to forward-model assumptions, we performed additional simulations incorporating source-space mismatch, forward-model mismatch, and more realistic noise conditions. While source-space mismatch produced only modest reductions in reconstruction accuracy, simulations generated using a BEM forward model and reconstructed using the single-shell model employed throughout this study showed larger performance reductions, particularly at lower SNRs and higher co-registration errors. Nevertheless, the qualitative dependence of reconstruction accuracy on SNR and co-registration precision remained unchanged across all robustness analyses. These findings suggest that the principal conclusions are reasonably robust to moderate model mismatch while also highlighting forward-model accuracy as a primary determinant of achievable laminar precision in empirical data. Future work could improve laminar inference precision by employing more sophisticated forward models, such as BEM or finite element models (FEMs). Targeted sensory paradigms that preferentially engage specific cortical laminae may also provide an important avenue for further validation of laminar specificity.

The sensitivity of laminar inference to orientation errors suggests that future improvements may come from more realistic models of cortical column geometry. In the present framework, corresponding vertices across layers share a common orientation,

but cortical columns are not strictly straight[56]. Future models could therefore incorporate depth-varying orientation vectors derived from equivolumetric laminar surfaces[40], as opposed to the equidistant surfaces used here. Estimating cortical column orientation using diffusion tensor imaging (DTI) is a promising alternative, as anisotropic diffusion within cortical gray matter can reflect the organization of cortical columns[57]. However, DTI-based estimates can be confounded by white matter fibers projecting into the cortex (the gyral bias effect), potentially biasing orientation measures. Advanced diffusion imaging techniques such as high angular resolution diffusion imaging (HARDI) and constrained spherical deconvolution, combined with surface-based analyses that sample diffusion metrics along radial cortical trajectories, can help isolate cortical diffusion signals from adjacent white matter influences[58,59]. Integrating these advanced imaging approaches into laminar MEG source models could yield more biologically plausible dipole orientation estimates and potentially improve depth localization, especially in highly folded cortex. The current framework provides a foundation for evaluating such models and for testing how column curvature influences laminar MEG source reconstructions.

Despite the significant improvements offered by our multilayer approach, a key limitation is the inability of current model-based inference methods to resolve two simultaneous sources of equal magnitude at different depths. Additional simulations including three weaker interfering sources at separate cortical locations produced only modest reductions in reconstruction accuracy, suggesting that the framework is reasonably robust to low-level concurrent activity. However, the inability to resolve two simultaneous sources of comparable magnitude at different depths indicates that the current approach should be interpreted as identifying the dominant laminar contribution within a localized cortical region and time window, rather than independently resolving all simultaneously active laminar generators. Although exact temporal alignment and perfectly matched source magnitudes are unlikely in biological systems, this limitation highlights the constraints of relying solely on Bayesian model comparison. One potential solution would be to use sensors capable of measuring higher spatial-frequency components of the magnetic fields, such as optically pumped magnetometers (OPMs), which could increase sensitivity to subtle spatial differences between closely spaced sources[60]. Future refinements should explore both sensor technology advances and alternative inference strategies, such as Bayesian model averaging (BMA), which assigns probabilistic weights to multiple competing models rather than selecting a single best-fit model[61]. These combined approaches may enhance the simultaneous resolution of multiple sources, overcoming a fundamental limitation of the current winner-takes-all approach.

3. Authors have done a great job with pushing the approach forward through rigorous characterization of their method and putting together a sophisticated analytical and statistical pipeline. However, uncertainties remain for practical use. In their approach authors capitalize on the minute differences of leadfields across laminae. Still, assigning the location of sources that are represented in noisy recordings to individual laminae is very challenging. In real data, inaccuracies can arise from errors in the forward model

and errors in the estimation of cortical column orientation (and these errors are unknown to the researcher). In addition, authors show the effect of patch size on multilayer reconstruction accuracy. That alone can lead to a bias and wrong reconstruction since real patch size is unknown. Also, there is the trade-off between time resolution and SNR for evidence profiles when choosing the temporal window length. Given all these limitations I would encourage authors to rephrase some of their strong statements (title, abstract and main text) especially pertaining to the inference across six laminae. One example is:

> Line 390 „Accurate laminar inference therefore requires a coregistration error of 2 mm or less, though a coarse deep-versus-superficial distinction may be possible in some locations at errors up to 5 mm, provided there is sufficient SNR.“

Again, this is for the case of white noise, a single source and no error in the forward model and does probably not hold for real data.

Still, my overall assessment of the manuscript is positive. Group statistics (as used here) can partly protect against some unsystematic errors. Maybe it is even possible to use strong sensory stimuli to generate high-SNR signals as a quality control or reference data (or for some kind of calibration) to gauge individual reconstruction accuracy (similar to a localizer in fMRI) in the same session as your paradigm of interest.

We agree that practical application of multilayer hpMEG requires careful consideration of uncertainties arising from forward-model inaccuracies, cortical-column orientation estimation, patch-size specification, temporal-window selection, and other factors that are difficult to quantify directly in empirical data. In response to these concerns, we have substantially expanded the simulation framework to assess robustness to more realistic noise structure (pink noise), concurrent neural activity, source-space mismatch, and forward-model mismatch (AGM and BEM simulations). These analyses demonstrate that the principal conclusions remain qualitatively unchanged, while also highlighting that accurate forward modeling is a major determinant of reconstruction performance. We have therefore revised the manuscript throughout to distinguish more clearly between theoretical feasibility demonstrated in simulations and empirical evidence for laminar-family-level dynamics.

We also agree that future applications would benefit from subject-specific quality-control procedures. We have added discussion of the potential use of dedicated localizer paradigms (e.g., robust visual or sensorimotor evoked responses with well-characterized laminar activation motifs) to assess achievable signal quality and identify cortical locations with favorable conditions for laminar inference within individual recording sessions. Such measurements could provide practical quality-assurance metrics for future studies and help constrain subsequent task-related analyses (lines 674-686):

An important challenge for future applications of multilayer hpMEG is the development of subject-specific quality-control procedures. Although our simulations define general requirements for reliable laminar inference, the achievable accuracy in any individual

dataset will depend on factors that are difficult to quantify directly, including forward-model accuracy, cortical column orientation estimates, co-registration precision, and local anatomical properties. One promising approach would be to incorporate dedicated localizer paradigms within the same recording session. For example, robust visual or sensorimotor evoked responses with well-characterized laminar activation sequences could be used to identify cortical locations with favorable anatomical and biophysical properties for laminar inference, quantify achievable signal-to-noise ratios, and constrain subsequent analyses of task-related activity. Such subject-specific reference measurements may provide a practical means of assessing the reliability of laminar inference in individual participants and of tailoring acquisition and analysis strategies to maximize reconstruction fidelity.

Reviewer #3

In this work, Szul et al. extend previous work of the group by combining high-precision MEG with fine-grained source models and Bayesian model comparison, moving from the differentiation of deep and superficial cortical sources to depth-resolved inference across all six cortical laminae.

Laminar inference of non-invasive electrophysiology in humans is of high relevance, and moving beyond the deep-superficial dichotomy to full cortical depth resolution is an important step forward in investigating lamina-specific neural dynamics in human cognition. The authors conduct extensive simulations to support the validity of their approach, incorporate post mortem atlases of laminar widths for laminar boundary estimates, and importantly demonstrate the method's applicability to empirical data by reconstructing laminar activity profiles from visual and motor event-related fields, with convincing results. Remaining challenges, such as differentiating multiple sources at different cortical depths, misestimation of patch sizes, and the restriction to a single pre-selected cortical patch location, are clearly delineated in the manuscript.

The present study should be of interest to cognitive and computational neuroscientists seeking to understand cortical computations across layers, as well as clinicians working on diseases with laminar vulnerabilities, such as Parkinson's and Huntington's disease.

General comments

1. While the manuscript is very clear and well written, the methods descriptions could be improved for clarity, as detailed below.

   We thank the reviewer for this positive assessment and for highlighting areas where the Methods could be clarified. In response, we have substantially revised the Methods section to improve transparency and readability. Specifically, we have clarified the definitions and computation of reconstruction error and bias, made explicit the distinction between continuous and binarized error measures used in different analyses, and provided more detailed descriptions of the statistical procedures (including permutation testing, bootstrap resampling, and effect size estimation). We have also clarified the temporal analysis framework (including the use of peak-centered versus

sliding windows), the definition of diagonal dominance, and the procedure used to determine optimal anatomical thresholds. Finally, we have streamlined and harmonized methodological descriptions across datasets to reduce redundancy and improve consistency.

2. The experimental findings should be discussed in light of the patch size sensitivity and the limitations of laminar inference for multiple simultaneous sources demonstrated in the simulations. Were different patch sizes tested? Conceptually, I wonder how one would select a patch size for sources known to extend across several vertices?

We agree that patch size is an important consideration for laminar source reconstruction because cortical depth and spatial extent are partially confounded. The submitted manuscript already included simulations demonstrating that patch-size mismatch systematically biases laminar inference, with underestimation of source extent producing a deep bias and overestimation producing a superficial bias (Supplementary Figure 17), consistent with previous hpMEG studies (Troebinger et al., 2014; Bonaiuto et al., 2018; Bonaiuto et al., 2021). To assess whether the empirical findings depended on the choice of patch size, we repeated the VEF, MF, and MEF-I analyses using patch sizes of 2.5, 5, 7.5, and 10 mm (Supplementary Figure 18). Across all datasets, the principal laminar findings remained qualitatively stable. In the visual dataset, the dominant granular and supragranular responses were preserved across all tested patch sizes. Similarly, the MF and MEF-I components exhibited highly similar laminar profiles, with only minor differences in the statistical significance of some supragranular effects at the smallest tested patch size.

These findings suggest that the reported laminar dynamics are not driven by a narrowly tuned spatial prior. However, we agree that patch size raises an important conceptual issue. Because cortical depth and spatial extent cannot be completely dissociated, an alternative interpretation of some apparent depth changes is that activity remains within the same laminae while varying in spatial extent over time. We have expanded the Discussion to explicitly acknowledge this possibility (lines 595-615):

A methodological consideration is the spatial extent of the underlying cortical generator. As shown in our simulations and previous hpMEG studies[19,20], mismatches between the true spatial extent of cortical activity and the patch size assumed during source reconstruction can systematically bias laminar inference. Specifically, underestimation of source extent biases solutions toward deeper layers, whereas overestimation biases solutions toward superficial layers (Supplementary Fig. 17). These findings highlight a fundamental limitation of current non-invasive laminar inference approaches: cortical depth and spatial extent are partially confounded. Importantly, however, the principal empirical findings proved largely robust across patch sizes ranging from 2.5 to 10 mm (Supplementary Fig. 18). In the visual dataset, the dominant granular and supragranular responses remained evident across all tested patch sizes. The MF and MEF-I components exhibited highly similar laminar profiles despite minor differences in the significance of some supragranular effects. This suggests that the reported results are not driven by a particular choice of patch size. Nonetheless, an alternative interpretation

of apparent changes in laminar dominance is that activity remains within the same laminar compartment while varying in spatial extent over time. Based on invasive recordings demonstrating layer-specific recruitment during visual processing and computational models predicting distinct laminar contributions to sensory and sensorimotor evoked responses[47,48,52–55], we consider genuine changes in laminar engagement a plausible explanation for the observed dynamics. However, current non-invasive methods cannot fully dissociate changes in depth from changes in spatial extent, and the observed dynamics may reflect a combination of both processes.

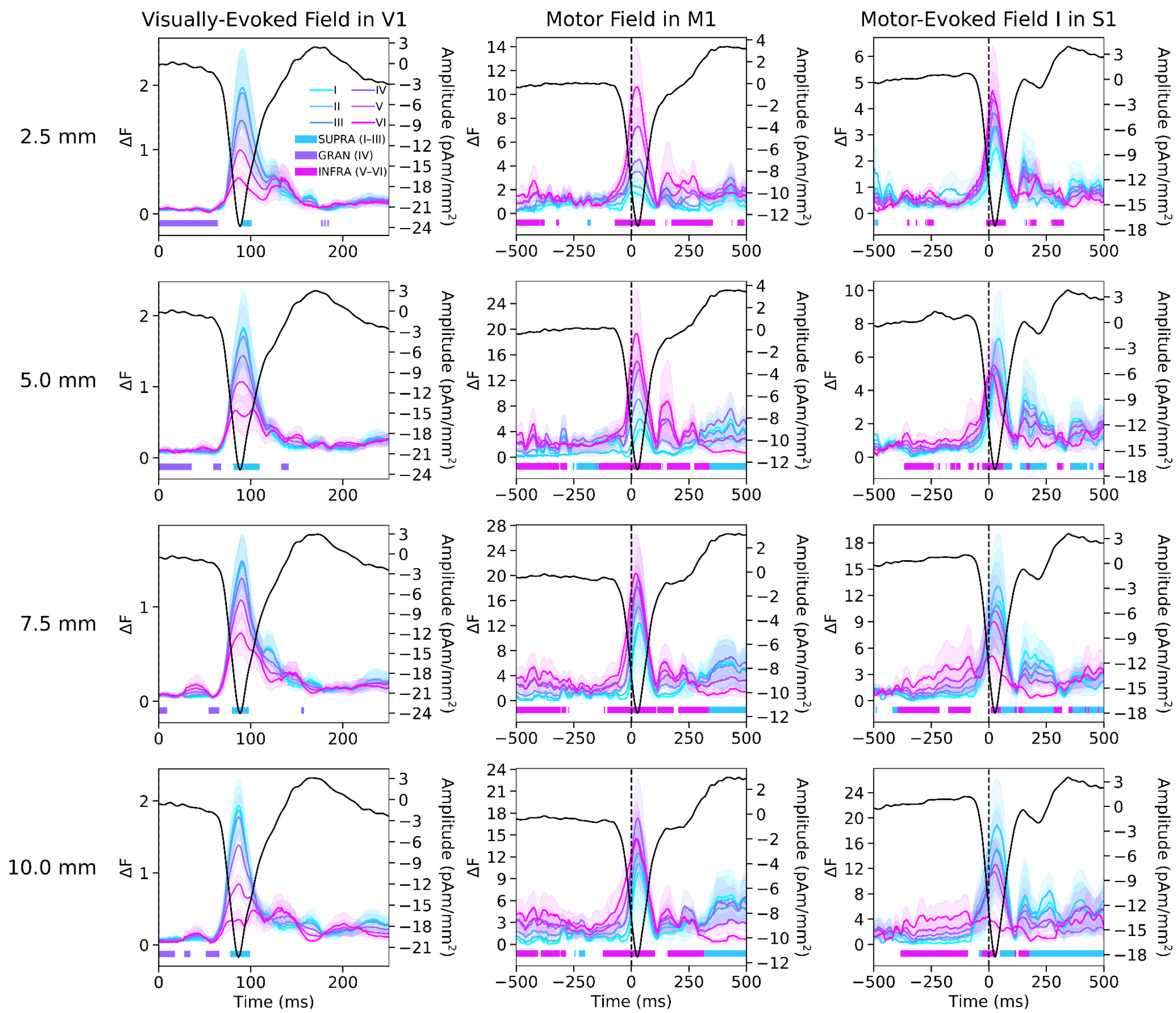

Supplementary Figure 18. **Sensitivity of empirical laminar inference to MSP patch size.** Sliding-window laminar inference results for the visually evoked field (VEF), motor field (MF), and motor-evoked field I (MEF-I; columns) using MSP patch sizes of 2.5 mm, 5 mm, 7.5 mm, and 10 mm (rows). Colored traces show lamina-specific relative model evidence (ΔF, relative to the worst model at each time point) averaged across participants, with shaded regions denoting SEM. Dark traces show the corresponding middle-layer source time series. Horizontal color bars indicate, at each time point, the laminar family with the highest protected exceedance probability (PEP), grouping layers into supragranular (laminae I-III), granular (lamina IV), and infragranular (laminae V-VI) families. Across all tested patch sizes, the principal laminar dynamics remained qualitatively stable. In the visual dataset, dominant granular and supragranular activity was preserved across patch sizes, whereas in the MF and MEF-I components the characteristic infra-/supragranular dynamics were largely unchanged, with only minor differences in the statistical significance and

temporal extent of some supragranular effects. These results indicate that the empirical findings are robust to plausible variation in the assumed spatial extent of cortical sources.

3. In general, I would have found more subtitles in the figures helpful. Having to constantly refer back to the legend to read a figure can be tedious. This is only a suggestion.

   This is an excellent suggestion. We have added subtitles to guide the reader.

Specific comments

1. Lines 150f: Might operationalizing the "error level" as the maximum fiducial displacement lead to a significant overestimation of co-registration error?

   We agree that defining co-registration error in terms of maximum fiducial displacement may overestimate the effective error relative to more distributed or temporally varying misalignment. Simulating realistic head movement and its correction is non-trivial and was beyond the scope of the present study. Our intention was to provide a simple and interpretable upper-bound measure of co-registration error. In this sense, the reported thresholds should be viewed as conservative: if laminar inference is achievable under these conditions, it is likely to remain feasible under more distributed or partially corrected misalignment. We have clarified this point in the manuscript and now explicitly state that our simulations do not model dynamic head movement or its correction (lines 837-841):

   This operationalization provides a simple and well-controlled measure of co-registration error, but may overestimate the effective misalignment relative to more spatially distributed or temporally varying head movement. Because realistic head motion and its correction were not explicitly simulated, the reported error levels should be interpreted as conservative upper bounds.

2. Lines 218ff: The definitions of reconstruction error and bias could be clearer: these appear to be averaged values, but it is not immediately obvious from the methods section what is averaged across (locations, depths, etc.) or normalized to what. Some of these details appear later in the results section but should be moved to the methods section for better readability. Additionally, an explanatory sentence addressing why reconstruction errors and biases are observed even though peak relative model evidences all lie on the diagonal (Figures 1f, g, and h) would be helpful.

   We thank the reviewer for this helpful suggestion. We agree that the definitions of reconstruction error and bias were not sufficiently explicit in the Methods. We have revised this section to clarify how these quantities are computed, including what is averaged and how values are expressed. Specifically, reconstruction error was computed at each cortical vertex as the absolute difference between the simulated and inferred depth (in layer index), and bias as the signed difference. These quantities were then averaged across simulated depths and vertices to obtain summary measures. For analyses expressed in millimeters, layer indices were scaled by local cortical thickness.

We have also clarified why non-zero reconstruction error and bias can be observed even when peak model evidence lies on the diagonal. While peak model evidence identifies the most likely depth for each simulated source, reconstruction error reflects the full distribution of inferred depths across simulations and locations. Small deviations in peak selection across vertices, particularly at lower SNR or in anatomically less favorable regions, contribute to non-zero average error and bias despite an overall diagonal structure in the mean model evidence matrices. These clarifications have been added to the Methods section (lines 928-939):

We quantified reconstruction accuracy by identifying, for each simulated cortical depth and vertex, the surface with the highest model evidence and comparing its depth to the known simulated source depth. Reconstruction error was defined as the absolute difference between the simulated and inferred depths (Figure 1g), measured both in number of surfaces and in millimeters by scaling surface indices using local cortical thickness. Reconstruction bias was defined as the signed difference between simulated and inferred depths (Figure 1h). These quantities were first computed per vertex and simulated depth, and then averaged across depths and cortical locations to obtain summary measures. Note that while mean model evidence matrices often exhibit a diagonal structure, indicating correct depth identification on average, reconstruction error and bias capture variability at the level of individual vertices and simulations, and are therefore sensitive to deviations that are not apparent in the averaged matrices.

3. Lines 251ff: For the analysis of how anatomical features determine the accuracy of laminar inference, I would have expected a mediation analysis, given that we know that dipole orientation relative to the scalp, scalp-to-cortex distance and cortical thickness all impact the leadfield difference. Please comment.

   We thank the reviewer for this insightful suggestion. We agree that mediation analysis provides a useful framework for understanding how anatomical factors influence laminar inference accuracy via their effect on lead-field separability. To address this, we performed a mediation analysis in which lead-field difference was treated as a mediator between anatomical features (orientation, distance, thickness) and reconstruction accuracy. The results show that the effects of cortical thickness and scalp-to-cortex distance on laminar inference are largely mediated by lead-field separability, with significant indirect effects and minimal residual direct effects for distance. In contrast, dipole orientation exhibited a strong direct effect on accuracy, with weaker and non-significant mediation via lead-field difference. These findings support the interpretation that lead-field separability is the primary mechanism through which anatomical factors constrain laminar inference, while orientation additionally influences signal detectability independently of depth separability. We have described this analysis in the Methods section (lines 999-1007):

   To further characterize the relationships between anatomical features and laminar inference accuracy, we performed a mediation analysis treating lead-field difference as a mediator. For each anatomical feature (dipole orientation, scalp-to-cortex distance, and

cortical thickness), we estimated (i) a linear regression model relating the feature to lead-field difference, and (ii) a logistic regression model relating laminar inference accuracy (binary: zero vs. non-zero reconstruction error) to both the feature and lead-field difference. Indirect (mediated) effects were computed as the product of the regression coefficients linking the feature to the mediator and the mediator to the outcome. Confidence intervals for indirect effects were obtained using bootstrap resampling (5,000 iterations) over cortical vertices.

and incorporated the results into the Results section (lines 395-406).

To determine how anatomical features influence laminar inference accuracy, we performed a mediation analysis with lead-field difference as a mediator. Cortical thickness showed a strong indirect effect via lead-field separability (indirect effect = 0.49, 95% CI [0.46, 0.52]), alongside a residual direct effect ($c' = 0.67$), indicating partial mediation. Scalp-to-cortex distance exhibited a smaller but significant indirect effect (indirect effect = -0.08, 95% CI [-0.08, -0.07]) with negligible direct effect ($c' \approx 0$), consistent with near-complete mediation. In contrast, dipole orientation showed no significant indirect effect (indirect effect = 0.05, 95% CI [-0.01, 0.11]) but a strong direct effect ($c' = 1.51$), suggesting an additional contribution to signal detectability independent of depth separability. Cortical thickness and distance therefore influence laminar inference primarily through their effect on lead-field separability, whereas dipole orientation exerts a largely independent effect, reinforcing the central role of lead-field separability as the primary determinant of laminar inference accuracy.

4. Lines 260ff: “These features were then related to reconstruction error using kernel density estimation and receiver operating characteristic (ROC) analysis to identify anatomical thresholds predictive of accurate inference.” I found this confusing. Earlier in the text (line 252ff), we learn that: “For each vertex, we computed absolute reconstruction error in millimeters. We then analyzed the relationship between reconstruction error and several anatomical properties.” From this, I would have expected reconstruction error to be a continuous variable but later sections of the manuscript suggest it is treated as a binarized measure - no reconstruction error vs. any reconstruction error. This should be made explicit in the manuscript.
   How does this reconcile with the four groups displayed in the marginal histograms of Fig. 7d,f,h? How are those defined?

   This was indeed not clear. Reconstruction error was indeed initially treated as a continuous variable (absolute error in millimeters) when characterizing spatial variation in laminar inference accuracy. However, for ROC analysis, error was binarized into zero vs. non-zero error to identify anatomical features predictive of correct versus incorrect layer assignment. This binarization was motivated by the highly skewed distribution of reconstruction error, with exact recovery (zero error) occurring in the majority of cortical locations (~85% of vertices under the simulated conditions). As a result, distinguishing correct from incorrect reconstructions provides a more interpretable criterion for identifying anatomical constraints. The four groups shown in the marginal histograms of

Fig. 7d,f,h are used solely for visualization and were not used in any statistical analysis. Specifically, vertices were partitioned into one group with zero reconstruction error and three additional groups defined by equally spaced bins spanning the range of non-zero errors (from the minimum to maximum observed error). This binning was used only to visualize how anatomical features vary across increasing levels of reconstruction error. We have revised the manuscript to clarify the distinction between continuous and binarized error measures in the Methods section (lines 974-991):

To evaluate how local anatomy influences laminar inference, we quantified reconstruction error at each cortical location and related it to anatomical features. For each vertex, we computed absolute reconstruction error in millimeters. Given the highly skewed distribution of errors, with exact recovery (zero error) occurring in the majority of cortical locations, we additionally defined a binary error variable (zero vs. non-zero error) for classification-based analyses. This binary variable was used in receiver operating characteristic (ROC) analyses to identify anatomical features predictive of correct versus incorrect laminar inference. Continuous error values were retained for descriptive analyses.

We then analyzed the relationship between binary reconstruction error and several anatomical properties: cortical thickness (measured between the pial and white matter surfaces), distance to the scalp surface, orientation of the cortical column relative to the scalp, and variability in lead field magnitude across depth. Orientation was computed as the absolute dot product between the simulated dipole orientation and the scalp normal at each vertex. Lead field variability was quantified as the root mean square difference in lead field magnitude across surface layers, relative to the superficial (pial) surface. These features were then related to binary reconstruction error using kernel density estimation and receiver operating characteristic (ROC) analysis to identify anatomical thresholds predictive of accurate inference.

and we have included a description of how the groups in the marginal histograms were defined in the caption of Figure 8 :

Figure 8. **Anatomical factors influencing laminar reconstruction error. a-b** Inflated-surface maps of laminar reconstruction error (a) and lead-field RMS difference (deep vs. superficial; b) **c-h** Inflated-surface maps and joint density and marginal histograms for each anatomical metric versus lead-field RMS difference: and dipole orientation (c,d), scalp-to-cortex distance (e,f), and cortical thickness (g,h). Joint density plots are color-coded by laminar reconstruction error (mm), and the marginal histograms group vertices into zero-error and three equally spaced bins spanning the range of non-zero errors. Lower lead-field differences, tangential orientations, greater distance to the scalp, and thinner cortex are associated with increased laminar-reconstruction error. Optimal thresholds in panels d, f, and h are indicated by black dashed lines and were defined using receiver operating characteristic (ROC) analysis (maximizing Youden's J statistic) to discriminate zero versus non-zero reconstruction error.

5. Lines 206f: "Because each time window is evaluated independently in this framework, a single 50 ms window was sufficient for our analysis". I am not sure I understand this sentence. A single time window in opposition to what?

We agree that this was ambiguous. Our intention was to convey that, in the simulation analyses, laminar inference was evaluated at the known peak of the simulated response, rather than across a full time course. Because the ground-truth temporal profile was known, it was not necessary to combine information across multiple time windows to identify the response peak. Instead, inference was performed using a single analysis window centered on this peak. We also note that robustness to window length was assessed using sliding time windows of varying durations (15-100 ms; Supplementary Figure 1), rather than a single fixed window, and this was not clearly stated in the original text. We have revised the manuscript to clarify both points (lines 913-921):

This was done to test the feasibility of a sliding time window approach previously applied to bilaminar MEG dynamics[25]. Because source reconstruction and model comparison is performed in each time window independently in this sliding time window framework, laminar inference was evaluated within a 50 ms window centered on the known peak of the simulated response, rather than across the full time course. We confirmed the robustness of this window size in supplementary simulations using sliding time windows of varying lengths (15, 25, 50, and 100 ms; Supplementary Fig. 1) across the whole epoch, which showed consistent reconstruction performance across a broad temporal range.

6. Lines 227ff: “Effect sizes were computed using Cohen’s d, defined as the difference between observed and shuffled means divided by the standard deviation of the shuffled distribution. We conducted bootstrap resampling (5,000 iterations), drawing random subsets of the data to compute 95% confidence intervals for error and bias.”
   Is calling this Cohen’s d appropriate? The definition of means differs between the observed mean and the shuffled mean and standard deviations are not pooled. Perhaps simply omit, i.e., “Effect sizes were defined as the difference”. Please define random subsets.

We agree that calling this quantity Cohen’s d was imprecise, because the denominator is the standard deviation of the permutation distribution rather than a pooled standard deviation from two samples. We have therefore removed the term Cohen’s d and now refer to this value as a standardized difference relative to the shuffled null distribution. We have also clarified the bootstrap procedure. For each SNR or co-registration level, reconstruction error and bias were first averaged across simulated depths within each cortical location. Bootstrap confidence intervals were then computed by resampling cortical locations with replacement and recomputing the mean error or bias across the resampled locations (lines 940-951):

To assess statistical significance, we performed permutation tests using 10,000 iterations, shuffling the free energy matrices for each simulated cortical depth and recomputing error and bias distributions under the null hypothesis of no depth sensitivity. P-values were calculated by comparing observed reconstruction errors and biases against these shuffled distributions, with statistical significance determined at $p < 0.05$.

Effect sizes were defined as standardized differences relative to the shuffled null distribution, computed as the difference between the observed mean and the mean of the shuffled distribution divided by the standard deviation of the shuffled distribution. We computed 95% confidence intervals for reconstruction error and bias using bootstrap resampling (5,000 iterations). For each SNR or co-registration error level, error and bias were first averaged across simulated depths within each cortical location; cortical locations were then resampled with replacement and the mean error or bias was recomputed for each bootstrap sample.

7. Lines 232f: "...the ratio of diagonal values to total values in the free energy matrix.."
   Do you mean the ratio of mean diagonal and off-diagonal values?

   We thank the reviewer for highlighting this ambiguity. The diagonal dominance score was defined as the proportion of total matrix mass accounted for by the diagonal entries, computed as the sum of diagonal elements divided by the sum of all elements in the matrix. This provides a measure of the extent to which model evidence is concentrated along the correct (diagonal) entries. We have revised the manuscript to clarify this definition (lines 952-957):

   We also computed a diagonal dominance score to quantify the extent to which model evidence matrices favored reconstructions at the correct depth. This score was defined as the proportion of total matrix mass accounted for by the diagonal values (i.e., the sum of diagonal values divided by the sum of all values in the free energy matrix). This metric was compared against a permuted distribution (*N* = 10,000) to assess whether observed diagonality exceeded chance levels.

8. Lines 339ff: "However, the probability density of surfaces with the maximal free energy demonstrates that in most locations, the peak free energy corresponded to either the most superficial or the deepest surface model."
   Out of curiosity: do you have an explanation for why this is the case? Why the most superficial or deepest surfaces?

   The observed tendency for peak model evidence to favor either the most superficial or deepest surfaces at low SNR arises from the structure of the MEG forward model and the depth-dependent separability of lead fields. Specifically, superficial and deep sources produce the most distinct sensor-level field patterns, whereas intermediate depths generate more similar (and thus less discriminable) lead fields. As a result, when the data are dominated by noise, model comparison tends to favor these "extreme" models because they maximize differences in predicted sensor patterns, even in the absence of true signal.

   To quantify this effect, we computed the mean cosine dissimilarity between the lead fields of each cortical depth and all other depths. This analysis revealed a clear U-shaped profile across cortical depth, with the most superficial and deepest layers exhibiting the greatest mean dissimilarity from the remaining layers, whereas intermediate depths showed substantially lower separability. This supports the

interpretation that extreme-depth models are preferentially selected under noisy conditions because their sensor-level lead fields are intrinsically more distinguishable.

This interpretation is also consistent with our anatomical analyses showing that laminar inference accuracy is strongly constrained by lead-field dissimilarity across depth, with reduced discriminability leading to increased reconstruction ambiguity. Importantly, the edge bias disappears as SNR increases, at which point the full diagonal structure of the model evidence matrix is recovered. We have added clarification in the manuscript to make this interpretation explicit (lines 154-162):

At low levels of SNR (< -50 dB), there was no pattern in the mean free energy (averaged over all simulated locations) over simulated cortical depths (Figure 2a). However, the probability density of peak model evidence showed a bias toward either the most superficial or the deepest surface model (Figure 2e). This likely reflects the greater separability of their lead fields at the sensor level: superficial and deep sources produce more distinct field patterns than intermediate depths, making them preferentially selected under noisy conditions. Consistent with this interpretation, lead-field dissimilarity exhibited a U-shaped profile across cortical depth, with the most superficial and deepest layers showing the greatest separability from the remaining layers (Supplementary Fig. 2).

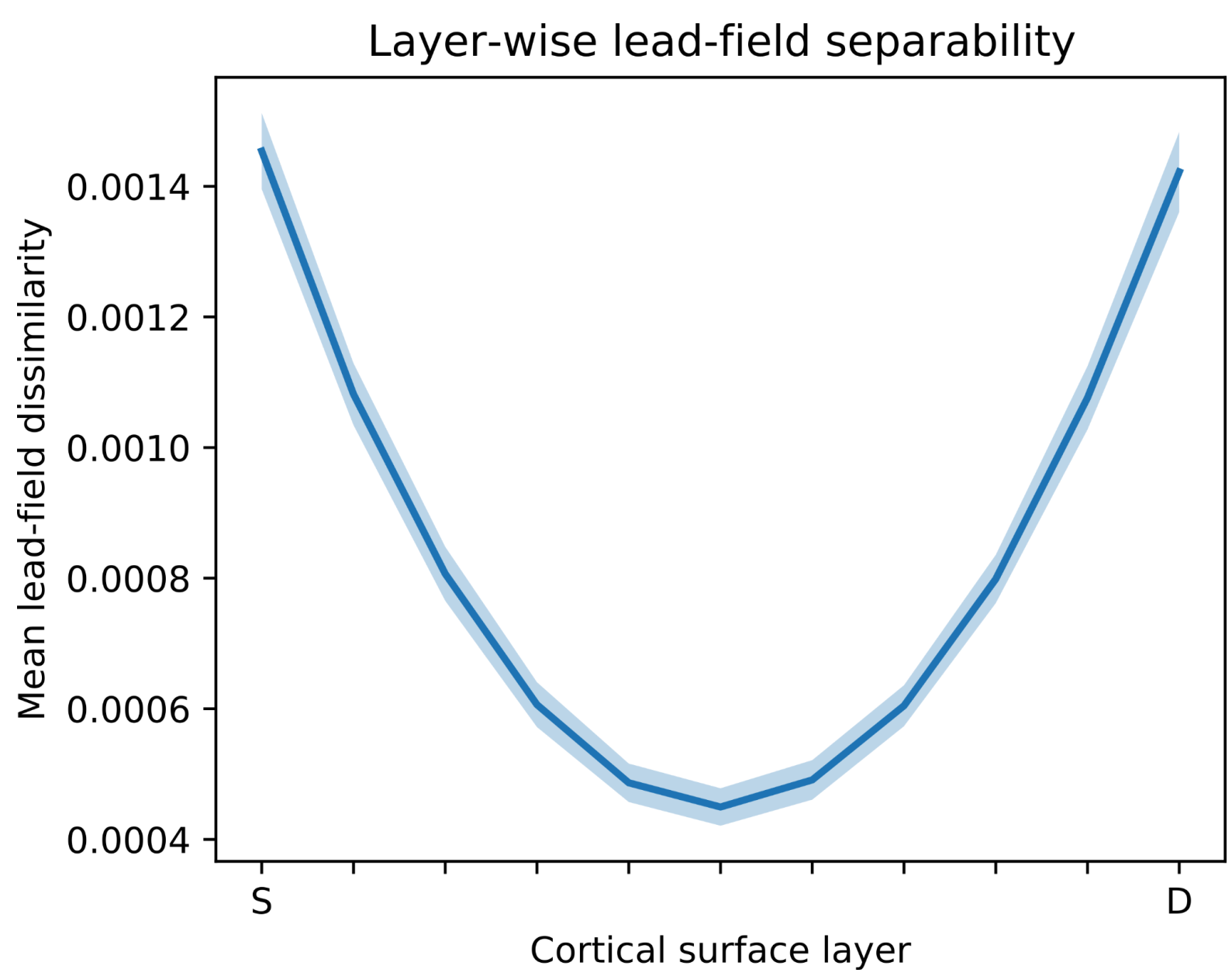

Supplementary Figure 2. **Lead-field separability across cortical depth.** Mean lead-field separability as a function of cortical surface layer, averaged across all simulated cortical locations. For each cortical vertex, lead fields were extracted for all cortical depth surfaces and normalized to unit length. Pairwise cosine dissimilarities (1 - |cosine similarity|) were then computed between the lead fields of each layer and all other layers. Lead-field separability for a given layer was defined as the mean cosine dissimilarity between that layer's lead field and the lead fields of all remaining cortical depths. Higher values therefore indicate that a layer produces more distinct sensor-level field patterns relative to other depths. The resulting profile exhibits a characteristic U-shape, with the most superficial (S) and deepest (D) cortical surfaces showing greater separability than intermediate depths. Shaded regions indicate ± SEM across cortical

vertices.

9. Lines 437f: “The increased variability in middle laminae suggests that reconstruction accuracy is not uniform across the cortex, requiring inference strategies that consider regional differences in laminar architecture.”
   Is this not simply due to the fact that middle layers lie in the middle, while more superficial and deep sources are naturally constrained to have less variability, as they can only be biased in one direction? (There is no deeper layer than the deepest surface.)

   We agree that greater variability in middle laminae may partly reflect the bounded nature of the laminar depth axis: superficial and deep laminae can only be misclassified inward, whereas middle laminae can be misclassified in either direction. Our original phrasing over-interpreted this variability as evidence specifically for regional differences in laminar architecture. We have revised the text to acknowledge this geometric constraint and to state more conservatively that laminar thickness and position jointly influence reconstruction error (lines 261-264):

   The greater variability observed for middle laminae likely reflects both their position with the cortical depth axis, where errors can occur in either direction, and regional variation in laminar thickness. Reconstruction accuracy is therefore likely shaped by both geometric constraints and regional differences in laminar architecture.

10. Lines 513ff: How is the optimal threshold defined?

    The optimal threshold was defined using receiver operating characteristic (ROC) analysis as the value that maximizes Youden’s J statistic (sensitivity - specificity). Specifically, for each anatomical feature, we computed the ROC curve relating the feature values to binary reconstruction accuracy (error vs. no error), and selected the threshold that maximized the difference between true positive rate and false positive rate. To ensure consistent interpretation, feature polarity was adjusted where necessary such that higher values corresponded to better discrimination (i.e., AUC ≥ 0.5). We have clarified this definition in the Methods section (lines 988-993):

    These features were then related to binary reconstruction error using kernel density estimation and receiver operating characteristic (ROC) analysis to identify anatomical thresholds predictive of accurate inference. Optimal thresholds were defined as the values that maximized Youden’s *J* statistic (sensitivity - specificity) on the ROC curve, after ensuring consistent feature polarity (AUC ≥ 0.5).

11. Concerning the empirical results: it is commonly assumed that differences in log model evidence should exceed 3 to be considered significant, yet in the present work, statistics are performed on the (non-significant) model evidences. Could you please explain this to the reader?

This is indeed an important point to discuss. The commonly used threshold of ΔF > 3 is a useful heuristic for interpreting model evidence differences at the single-subject level, corresponding to strong evidence in favor of one model over another in a Bayesian model comparison framework. In the present study, however, we perform group-level statistical inference on model evidence values across participants using a hierarchical Bayesian framework (VBA), which evaluates consistency of model evidence differences across subjects. In this context, even sub-threshold differences at the individual level can contribute to reliable group-level effects if they are consistent across participants. We have clarified this distinction in the manuscript and now explicitly state that ΔF > 3 is not used as a significance threshold for group-level inference (lines 1063-1081):

Although differences in free energy (ΔF) greater than 3 are commonly interpreted as strong evidence in single-subject model comparisons, this heuristic does not extend to group-level inference. The ΔF > 3 criterion reflects within-subject evidence strength and cannot be used as a threshold for statistical significance across participants. In the present study, ΔF values were therefore used descriptively, and statistical inference was performed at the group level using a hierarchical Bayesian framework.

For Bayesian model selection we used the VBA (Variational Bayesian Analysis) toolbox[42]. The free energy measures of the six laminae were grouped in three families: supragranular (laminae I-III), granular (lamina IV) and infragranular (laminae V-VI) families. At each time point, family-wise random effects Bayesian model selection was applied to the subject-level free energy values. This approach treats model identity as a random variable across participants and estimates the population frequency of each laminar family, thereby accounting for inter-subject variability in model preference[43,44]. In the case when the posterior probability that all families are equally frequent in the population was below 0.5, we calculated protected exceedance probabilities (PEPs) to quantify the probability that one laminar family was more frequent in the population than the competing alternatives while accounting for chance-level differences in model frequency. Consequently, group-level inference was based on the consistency of model evidence across participants rather than on averaged free energy values.

Figures

1. Figure 1c is hard to read. I found Figure 1f confusing, as it does not seem to match the later depictions of "Mean model evidence (free energy, ΔF) matrices (row-normalized to the worst model" (legend of Figure 2). Figure 1f is clearly not row-normalized and uses percentage values instead of row-normalized delta Fs. In addition, I would suggest describing the methodological details in the main manuscript as well, not only in the figure legend.

   We have updated Figure 1 so that panel c is easier to read, and so that it correctly shows the traces in panel e as columns of panel f and no longer erroneously uses percentage values. We have also updated the caption of Figure 2 to replace "row-normalized to" with "relative to", in order to avoid confusion.

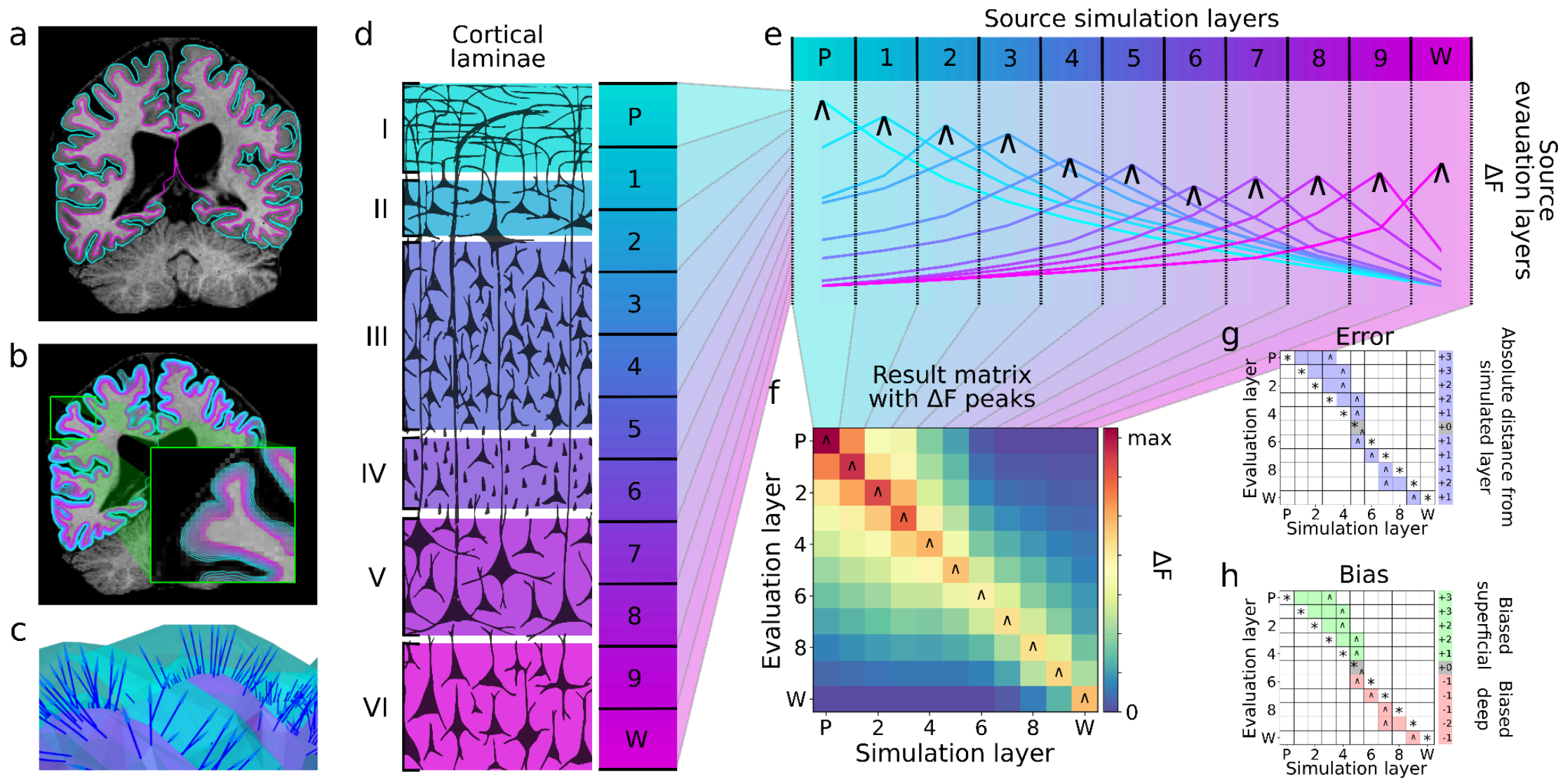

Figure 1. **Overview of the simulation strategy and analysis. a** Pial and white matter boundaries surfaces are extracted from anatomical magnetic resonance imaging (MRI) volumes. **b** Intermediate equidistant surfaces are generated between the pial and white matter surfaces (labeled as superficial (S) and deep (D) respectively). **c** Surfaces are downsampled together, maintaining vertex correspondence across layers. Dipole orientations are constrained using vectors linking corresponding vertices (link vectors; represented by blue lines). **d** The thickness of cortical laminae varies across the cortical depth, which is evenly sampled by the equidistant source surface layers. **e** Each colored line represents the model evidence (relative to the worst model, ΔF) over source layer models, for a signal simulated at a particular layer (the simulated layer is indicated by the line color). The source layer model with the maximal ΔF is indicated by a caret. **f** Result matrix summarizing ΔF across simulated source locations, with peak relative model evidence marked with a caret. **g** Error is calculated from the result matrix as the absolute distance in mm or layers from the simulated source (asterisk) to the peak ΔF (caret). **h** Bias is calculated as the relative position of the peak ΔF (caret) to the simulated source (asterisk) in layers or mm.

2. The colorbar legend in Figure 4 indicates that thickness is displayed in mm, but the text suggests that proportional thickness is shown. Please clarify. If thickness is indeed displayed in mm, please comment on the cortical thickness across layers, which would then appear to be too small, even for ex vivo data.

We thank the reviewer for catching this inconsistency. The Figure 4 surface maps display proportional laminar thickness derived from the BigBrain atlas, not absolute thickness in millimeters. The colorbar label was therefore incorrect. We have corrected the colorbar and figure legend to indicate that these values represent proportional cortical depth. Absolute laminar thickness in millimeters was computed separately by scaling proportional laminar boundaries by local cortical thickness for the reconstruction-error analyses.

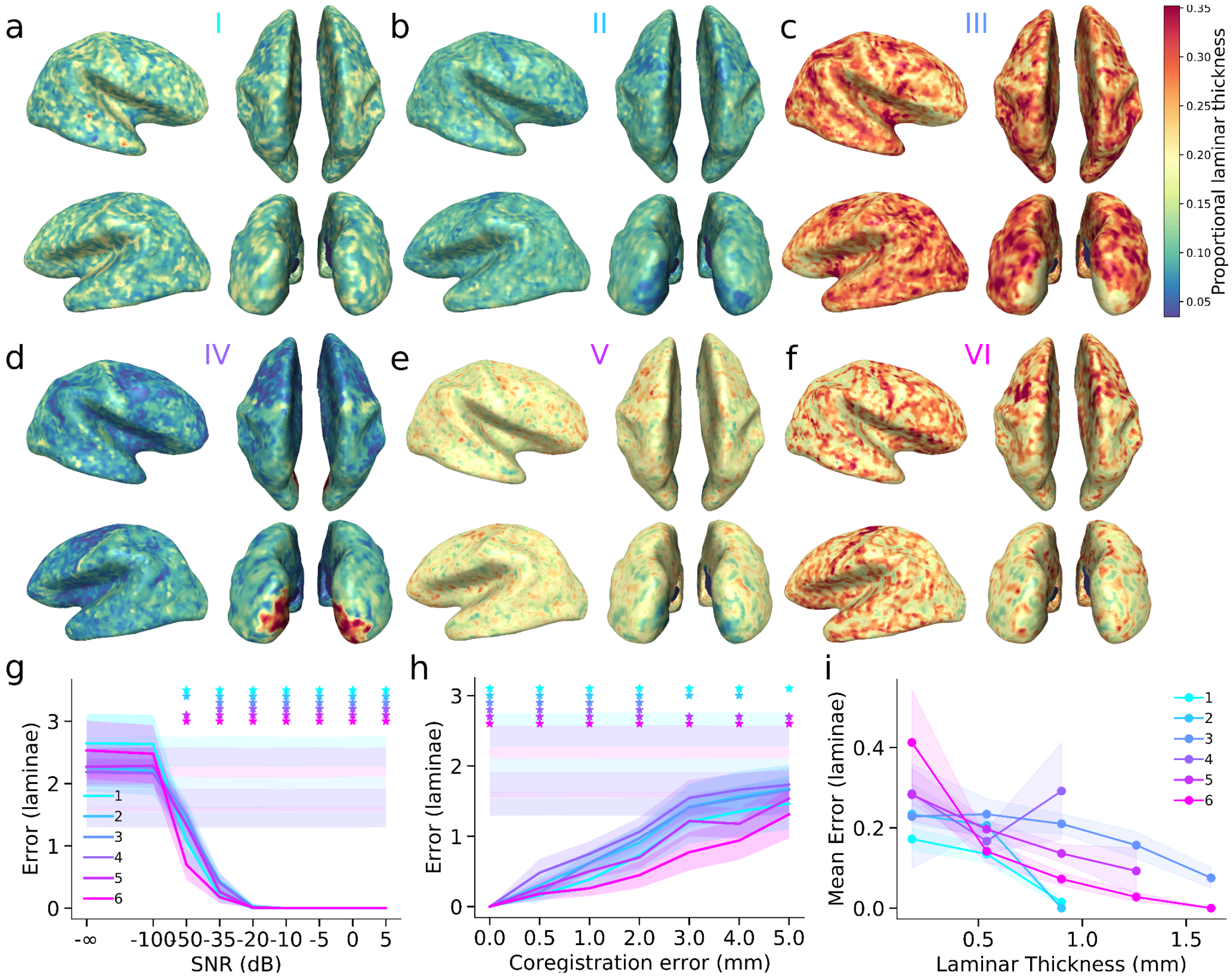

Figure 4. **Laminar source-reconstruction accuracy as a function of SNR, co-registration error, and cortical thickness. a-f** Cortical surfaces showing proportional laminar thickness across the brain, derived from the BigBrain atlas and mapped onto the surface models (representing laminae **I**-VI respectively) used in the simulations. These maps illustrate the variability of laminar thickness across cortical regions, which influences inference accuracy. Proportional laminar boundaries were subsequently scaled by local cortical thickness to compute laminar depths in millimeters for reconstruction-error analyses. **g** Mean laminar reconstruction error (in laminae) across SNR levels, computed as the absolute difference between the simulated and inferred lamina. Lines indicate the mean error for each lamina, with shaded bands representing 95% confidence intervals. Lighter shaded bands represent shuffled null distributions. Asterisks mark SNR levels where reconstruction error was significantly lower than chance. **h** Mean laminar reconstruction error as a function of co-registration error. **i** Relationship between laminar thickness and reconstruction error. Each line represents a different lamina (I-VI), showing how increased laminar thickness is associated with reduced mean reconstruction error (the shaded regions denote SEM).

3. Fig. 4i, 5c-h, 8 and 9: confidence intervals are missing.

We thank the reviewer for pointing this out. We have now added shaded regions representing the standard error of the mean (SEM) to these figures to better convey variability across participants and cortical locations. Figure legends have been updated accordingly.

4. Fig. 7: d,f,h: I think the y-axis label should read “Leadfield RMS difference” not “Leadfield RMSE”? Please mention the optimal threshold in the legend.

   We have corrected the y-axis label to “Leadfield RMS difference” and expanded the figure legend to clarify how the optimal thresholds were defined. Specifically, the dashed lines indicate thresholds derived from ROC analysis (maximizing Youden’s J statistic) for discriminating between zero versus non-zero reconstruction error. This is now explicitly stated in the legend:

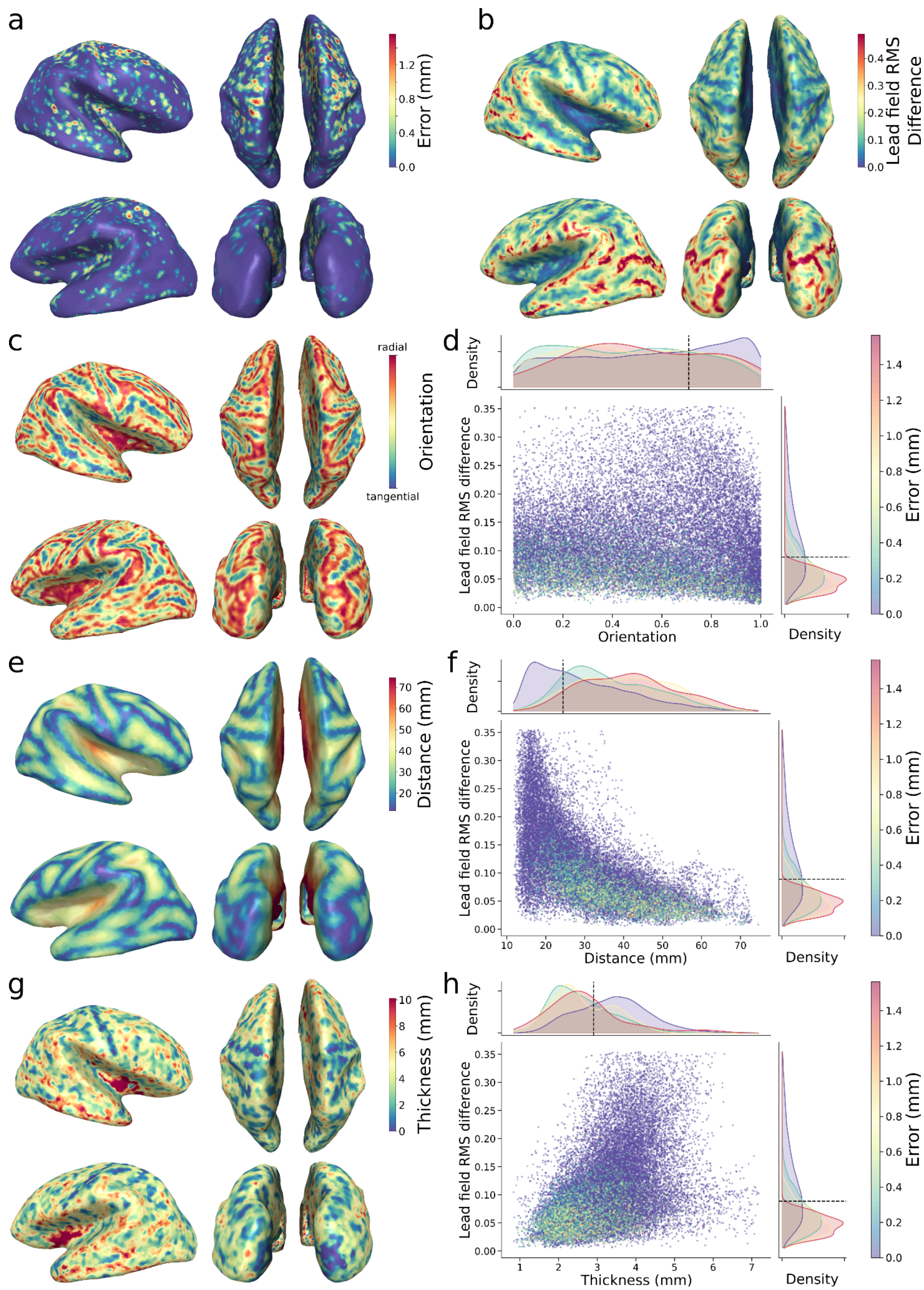

Figure 8. **Anatomical factors influencing laminar reconstruction error. a-b** Inflated-surface maps of laminar reconstruction error (a) and lead-field RMS difference (deep vs. superficial; b) **c-h** Inflated-surface maps and joint density and marginal histograms for each anatomical metric versus lead-field RMS difference: and dipole orientation (c,d), scalp-to-cortex distance (e,f), and cortical thickness (g,h). Joint density plots are color-coded by laminar reconstruction error (mm), and the marginal histograms group vertices into zero-error and three equally spaced bins spanning the range of non-zero errors. Lower lead-field differences, tangential orientations, greater distance to the scalp, and thinner cortex are associated with increased

laminar-reconstruction error. Optimal thresholds in panels d, f, and h are indicated by black dashed lines and were defined using receiver operating characteristic (ROC) analysis (maximizing Youden's J statistic) to discriminate zero versus non-zero reconstruction error.

Supplementary material

1. The shared preprocessing and analysis framework for the empirical data currently leads to substantial duplication of the methodological descriptions. This would be much easier to read (and differences between pipelines easier to spot) if later method descriptions referred back to earlier text and simply highlighted the differences. For example, there is extensive overlap between the last two paragraphs on page 3 and the first two on page 5. The CTF MEG system is referenced three times, each time with a different level of detail.

   We agree that the substantial overlap between the empirical dataset descriptions made it unnecessarily difficult to identify dataset-specific methodological differences. We have therefore revised the Supplementary Materials to streamline the presentation and reduce redundancy across sections. Specifically, we now use the visually cued button-press dataset as the reference preprocessing pipeline for the motor datasets, and the self-paced button-press dataset description now refers back to this pipeline while emphasizing only the methodological differences specific to that dataset (e.g., fiducial localization using 3D head scans, autoreject-based epoch repair, and differences in task structure). We also clarified why certain preprocessing steps differed across datasets, including the use of tSSS and cross-session realignment for the upright multi-session motor recordings, ICA-based artifact correction for motor-related artifacts, and eye-tracking-based trial rejection for the visually evoked dataset. In addition, we standardized the description of the CTF MEG acquisition system across all datasets for consistency.

2. The exclusion of 8 subjects with insufficient SNR at the response peak of the visually evoked response dataset should be moved from the supplementary material to the main manuscript.

   We thank the reviewer for this suggestion. We have moved this information to the Empirical Data Analysis subsection of the Methods section and clarified how SNR was computed and applied as an exclusion criterion (lines 1017-1025). We also note that the original manuscript incorrectly reported the exclusion of eight participants from the visually evoked response dataset. Following verification of the analysis pipeline and participant inclusion records, the correct number is six participants. This has been corrected throughout the manuscript and supplementary materials.

   All laminar inference analyses were performed on trial-averaged ERFs, rather than on single-trial data. Sensor-level SNR was therefore used as the primary measure of data quality, reflecting the reliability of the averaged response (Supplementary Fig. 14). Sensor-level SNR was computed within the analysis window as the ratio in dB of the RMS of the trial-averaged signal across channels to the RMS of trial-by-trial deviations from that mean, yielding a single SNR time series per participant. Participants whose

SNR within this window was < -15 dB were excluded (visually evoked response dataset: $N = 6$; visually cued button press dataset: $N = 0$; self-paced button press dataset: $N = 0$).

3. The high similarity of the responses in the two motor tasks seems overstated. I do not object to pooling the data, but would suggest toning down the claim of high replicability.

   We agree that our original wording overstated the degree of similarity between the two motor datasets. While the laminar dynamics are qualitatively consistent across tasks (e.g., early infragranular dominance in M1 and mixed infra-/supragranular responses in S1), there are clear differences in amplitude and timing characteristics between datasets. We have revised the manuscript throughout to describe these effects more cautiously as qualitatively similar or broadly consistent, rather than highly reproducible.

   Introduction (lines 102-104):

   In the somatosensory and motor cortices, analysis of two independent datasets reveals broadly consistent deep-superficial activation patterns characteristic of motor execution.

   Results (lines 456-459):

   Qualitative inspection revealed broadly similar laminar dynamics across the two datasets for both components (Supplementary Fig. 16), motivating their combination to enable group-level Bayesian model selection, which was not feasible within either dataset alone due to limited sample size.

   (lines 475-478):

   The qualitative correspondence between laminar dynamics observed in the two original datasets (Supplementary Fig. 16), together with their convergence in the combined analysis, supports the use of multilayer hpMEG to recover established sensorimotor activation motifs and extend them into the laminar domain.

   Discussion (lines 512-517):

   In motor circuits, combining two independent datasets revealed consistent laminar trends across datasets: motor fields in primary motor cortex were dominated by infragranular activity beginning before movement onset and recurring around execution, consistent with corticospinal output, followed by delayed supragranular responses, while motor-evoked responses in primary somatosensory cortex showed predominant superficial activity at movement onset, consistent with somatosensory reafference.

   Supplementary Figure 16 caption:

   Supplementary Figure 16. **Qualitatively consistent laminar activation patterns in motor and somatosensory cortices across tasks. a** Locations of the selected motor field (MF) source vertices for each participant in the visually cued button-press task, projected onto an inflated

fsaverage cortical surface; each colored sphere denotes one subject. **b** Sliding-window laminar inference for the MF in the visually cued button-press task. **c** Locations of the selected motor-evoked field I (MEFI) source vertices for each participant in the visually cued button-press task. **d** Sliding-window laminar inference for the MEFI in the visually cued button-press task. **e** Locations of the selected MF source vertices for each participant in the self-paced button-press task. **f** Sliding-window laminar inference for the MF in the self-paced button-press task. **g** Locations of the selected MEFI source vertices for each participant in the self-paced button-press task. **h** Sliding-window laminar inference for the MEFI in the self-paced button-press task. In b, d, f, and h, colored traces show lamina-specific relative model evidence (ΔF; shaded regions denote SEM) and black traces show the corresponding middle-layer source time series. Across both tasks and both cortical regions, qualitatively similar, though not identical, laminar dynamics are observed, with dominant infragranular activity surrounding movement onset in M1 followed by delayed supragranular activity, and predominantly infra- and then supra-granular responses in S1 at movement onset with additional superficial transients at later latencies.

Reviewer #1 (Remarks to the Author):

The revised manuscript presents a robust multilayer MEG source reconstruction framework designed for depth resolved laminar inference. The revision has adequately addressed the methodological and interpretative concerns raised during the initial review. The integration of systematic simulations alongside empirical validations across three independent datasets supports the reliability of the proposed framework. The transparent discussion of limitations regarding simultaneous source separation and forward model accuracy ensures appropriate boundary conditions for the findings. The manuscript is structurally sound, scientifically rigorous, and suitable for acceptance in its present form.
Looking ahead, extending this multilayer MEG source reconstruction framework to higher order complex cognitive processes, such as working memory, represents a highly valuable scientific trajectory. Furthermore, this technique holds significant potential for investigating predictive coding in psychosis. Current computational psychiatry holds that the core pathophysiology of conditions like schizophrenia involves dysregulation in the laminar specific routing of prediction error signals and precision weighting across cortical hierarchies. A noninvasive, time-resolved, depth-resolved laminar inference technique can provide the necessary empirical means to directly quantify these abnormal communications at specific cortical layers, thereby offering crucial neurophysiological evidence for the predictive coding dysfunction hypothesis.

Minor comment
Lines 1082-1084: The data repository link provided within the revision in the “Data availability” section (https://doi.org/10.5281/zenodo.20721185) is currently inaccessible (DOI not found). To facilitate a thorough evaluation and uphold research transparency, I encourage the authors to provide a secure reviewer access link for the preprocessed data.

We thank the reviewer for their positive assessment. The Zenodo repository has now been made publicly accessible and the Data Availability statement has been updated with the correct DOI.

Reviewer #2 (Remarks to the Author):

Authors have addressed my concerns and I am happy to recommend publication. The revision is very comprehensive and includes a lot of new computations and analysis. A small minor comment: In the revision, when discussing possible effects of additional sources, colored noise or different forward models, they conclude on several occasions: "Nevertheless, the overall structure of the results remained similar to the primary simulations, with reconstruction accuracy improving monotonically with increasing SNR and decreasing co-registration error (Supplementary Fig. 9 and 13)." A monotonic improvement of any performance measure with increasing SNR and decreasing co-registration is rather trivial and not very interesting. It is more interesting to conclude under what conditions accurate laminar inference is still possible. But overall, I am happy with this manuscript since all the data is clearly presented in the main text and Suppl Mat.

We thank the reviewer for this suggestion. We have revised the relevant sentences to emphasize the practical conditions under which accurate laminar inference remains possible, rather than simply noting the monotonic dependence on SNR and co-registration precision.

Reviewer #3 (Remarks to the Author):

I thank the authors for their thoughtful and thorough revision of the manuscript. The methods section has been substantially clarified and now provides a much clearer description of the experimental design and analytical approach. The extensive supplementary analyses the authors have added strengthen the robustness of their conclusions. I have no further concerns and am happy to recommend this manuscript for publication.

We thank the reviewer for their positive assessment and recommendation for publication.
